# Implementation and Outcomes of Virtual Care Across a Tertiary Cancer Center During COVID-19

**DOI:** 10.1001/jamaoncol.2020.6982

**Published:** 2021-01-07

**Authors:** Alejandro Berlin, Mike Lovas, Tran Truong, Sheena Melwani, Justin Liu, Zhihui Amy Liu, Adam Badzynski, Mary Beth Carpenter, Carl Virtanen, Lyndon Morley, Onil Bhattacharyya, Marnie Escaf, Lesley Moody, Avi Goldfarb, Luke Brzozowski, Joseph Cafazzo, Melvin L. K. Chua, A. Keith Stewart, Monika K. Krzyzanowska

**Affiliations:** 1 Smart Cancer Care Program, Princess Margaret Cancer Centre, University Health Network, Toronto, Ontario, Canada; 2 Data Science Program, Princess Margaret Cancer Centre, University Health Network, Toronto, Ontario, Canada; 3 Techna Institute, University Health Network, Toronto, Ontario, Canada; 4 Department of Radiation Oncology, Faculty of Medicine, University of Toronto, Toronto, Ontario, Canada; 5 Healthcare Human Factors, University Health Network, Toronto, Ontario, Canada; 6 Princess Margaret Cancer Centre, University Health Network, Toronto, Ontario, Canada; 7 Dalla Lana School of Public Health, University of Toronto, Toronto, Ontario, Canada; 8 University Health Network Digital, University Health Network, Toronto, Ontario, Canada; 9 Women’s College Hospital, Toronto, Ontario, Canada; 10 Rotman School of Management, University of Toronto, Toronto, Ontario, Canada; 11 eHealth Innovation, University Health Network, Toronto, Ontario, Canada; 12 Division of Radiation Oncology, National Cancer Centre Singapore, Singapore; 13 Division of Medical Sciences, National Cancer Centre Singapore, Singapore; 14 Oncology Academic Programme, Duke-NUS Medical School, Singapore; 15 Ontario Health, Cancer Care Ontario, Ontario, Canada; 16 The Cancer Quality Lab, Princess Margaret Cancer Centre, Toronto, Ontario, Canada

## Abstract

**Importance:**

The coronavirus disease 2019 (COVID-19) pandemic has burdened health care resources and disrupted care of patients with cancer. Virtual care (VC) represents a potential solution. However, few quantitative data support its rapid implementation and positive associations with service capacity and quality.

**Objective:**

To examine the outcomes of a cancer center–wide virtual care program in response to the COVID-19 pandemic.

**Design, Setting, and Participants:**

This cohort study applied a hospitalwide agile service design to map gaps and develop a customized digital solution to enable at-scale VC across a publicly funded comprehensive cancer center. Data were collected from a high-volume cancer center in Ontario, Canada, from March 23 to May 22, 2020.

**Main Outcomes and Measures:**

Outcome measures were care delivery volumes, quality of care, patient and practitioner experiences, and cost savings to patients.

**Results:**

The VC solution was developed and launched 12 days after the declaration of the COVID-19 pandemic. A total of 22 085 VC visits (mean, 514 visits per day) were conducted, comprising 68.4% (range, 18.8%-100%) of daily visits compared with 0.8% before launch (*P* < .001). Ambulatory clinic volumes recovered a month after deployment (3714-4091 patients per week), whereas chemotherapy and radiotherapy caseloads (1943-2461 patients per week) remained stable throughout. No changes in institutional or provincial quality-of-care indexes were observed. A total of 3791 surveys (3507 patients and 284 practitioners) were completed; 2207 patients (82%) and 92 practitioners (72%) indicated overall satisfaction with VC. The direct cost of this initiative was CAD$ 202 537, and displacement-related cost savings to patients totaled CAD$ 3 155 946.

**Conclusions and Relevance:**

These findings suggest that implementation of VC at scale at a high-volume cancer center may be feasible. An agile service design approach was able to preserve outpatient caseloads and maintain care quality, while rendering high patient and practitioner satisfaction. These findings may help guide the transformation of telemedicine in the post COVID-19 era.

## Key Points

**Question:**

Can virtual care (VC) be rapidly implemented across a tertiary center during the coronavirus disease 2019 (COVID-19) pandemic, and what are service capacity and quality outcomes?

**Findings:**

This cohort study of 22 085 VC visits at a single cancer center suggests feasibility of an agile service design process for implementation of VC at scale. This approach preserved outpatient caseloads and maintained care quality in all 6 care-quality domains of care quality laid out by the Institute of Medicine while rendering high patient and practitioner satisfaction.

**Meaning:**

These data support the value proposition of VC to safeguard system capacity, while minimizing the disruption to patient care during a pandemic.

## Teaser

This cohort study of a publicly funded comprehensive cancer center examines the outcomes of a cancer center–wide virtual care program in response to the COVID-19 pandemic.

## Introduction

Virtual care (VC) is the provision of medical care enabled by information and communication technologies when distance separates participants.^1^ Currently, VC has been selectively applied in the routine management of chronic conditions,^2^ including oncologic conditions. With the outbreak of the coronavirus disease 2019 (COVID-19) pandemic, there has been a systemic adoption of VC as a lever to encourage adherence to physical-distancing measures and limit interruptions in the delivery of ambulatory health care services.^3^ Patients with cancer may harbor higher risks of susceptibility to and mortality from COVID-19^4,5^; thus, minimizing patient risk of infection without disrupting critical oncologic care became a major imperative.

The Princess Margaret Cancer Centre (PM), University Health Network, Toronto, Ontario, Canada, conducts close to 2000 outpatient visits daily (approximately 1000 ambulatory clinics and approximately 1000 ambulatory treatments and procedures). On March 11, 2020, the PM executive board set the explicit goal of reducing in-person clinic visits by 50%. We report our experience with the implementation of a hospitalwide VC platform, which included simultaneous collection of longitudinal quantitative data on uptake across disciplines, quality-of-care indexes, and qualitative survey data from all stakeholders.

## Methods

**Figure 1.**
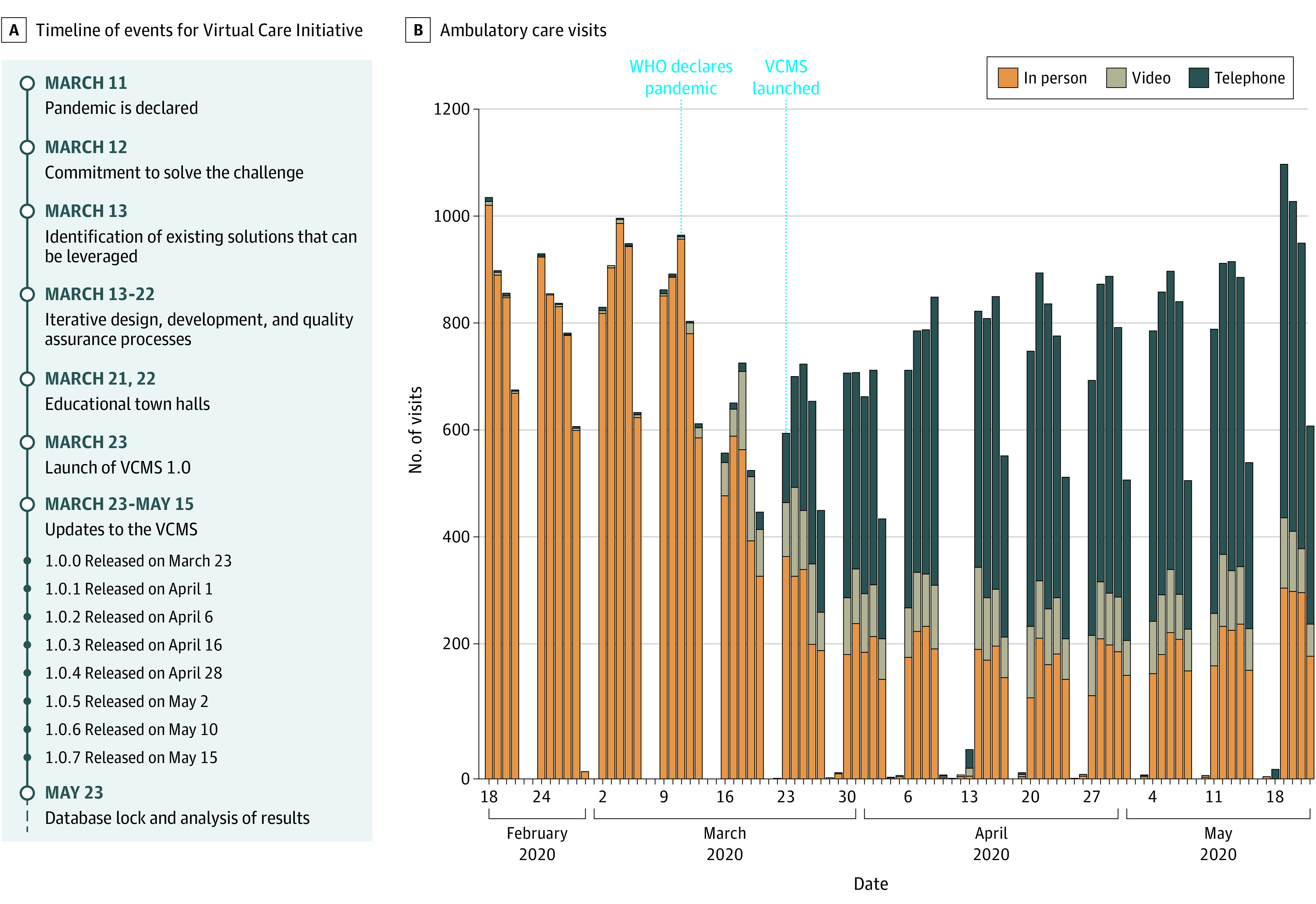
Timeline of the Virtual Care Initiative and Number of Ambulatory Visits Over Time Ambulatory clinics volumes from February 18 to May 22, 2020, were stratified by type of appointment (in-person and virtual care [telephone or video]) for every business day of the corresponding week. Dates of COVID-19 declaration of pandemic by the World Health Organization (WHO) and deployment of the Virtual Care Management System (VCMS) are highlighted in blue.

Agile service design mindset and methods^6^ were used to understand the current state of clinics at the PM with the aim of streamlining the mass redistribution of VC visits and supporting health care practitioner remote workflows. A description of this process is provided in the eMethods in the Supplement, along with the timeline of the initiative (Figure 1). Data were collected from the PM from March 23 to May 22, 2020. This study was reviewed and approved by the institutional review board of PM, including a waiver of written informed consent. This study followed the Strengthening the Reporting of Observational Studies in Epidemiology (STROBE) reporting guideline.

The resulting digital solution—Virtual Care Management System (VCMS)—is a browser-based application developed in house that integrates with the enterprise scheduling system (Pathways Healthcare Scheduling, McKesson Corp) and electronic medical record system (QuadraMed Corp). The main functionalities include (1) listing the physician’s upcoming in-person visits, federation of last clinical note for rapid contextualization, and documentation of the VC triage decision (keep in-person visit, reschedule to telephone or video appointment, or delay the visit), with decisions transferred to the administrative assistants in a task list for rebooking; and (2) VC clinic list with functionality to capture and communicate visit-specific medical orders to administrative staff (patient flow coordinators) to ensure timely, traceable, and coordinated completion.

The impact of this initiative was assessed in 3 domains: care delivery, patient and practitioner experiences with VC, and potential cost savings, including 6 domains of quality outlined by the Institute of Medicine: effectiveness, safety, timeliness, patient-centeredness, equitability, and efficiency.^7^

Data are summarized as number (percentages) for categorical variables and means (SDs) or medians (ranges) for continuous variables. The χ^2^ test was used for comparison of survey responses among VC modalities. To assess the association between sociodemographic factors and VC modality with patient survey response, multivariable ordinal logistic regression was used. Univariable ordinal logistic regression was performed to compare responses between the 2 rounds of surveys among practitioners. Observations with missing data in either responses or demographics were excluded. To account for intraparticipant correlation attributable to multiple surveys completed by the same individual, clustered robust SEs were used to calculate Wald test *P* values. The Brant test was used to assess proportionality assumption in the model. To assess whether the volume of ambulatory visits after VCMS deployment were restored to pre–COVID-19 levels, multivariable log-linear models were fitted to the daily number of visits (on log scale), adjusting for the number of visits in the previous day (on log scale) to account for the correlation over time. Weekends and statutory holidays were excluded because of low numbers.

All tests were 2-sided, and a threshold of *P* < .05 was set for statistical significance. All analyses were performed in the R statistical environment, version 3.5.2 (R Foundation for Statistical Computing). Additional assessment indexes and statistical analysis details are provided in the eMethods in the Supplement.

## Results

The VCMS was launched 12 days after the declaration of the COVID-19 pandemic (Figure 1). During the study period, adoption of VCMS reached 440 registered practitioners (76%). The total direct cost was CAD$ 202 537 (eTable 1 in the Supplement). Satisfaction specifically with VCMS was consistently high across users, with 82 physicians, 26 administrative assistants, and 24 patient flow coordinators being satisfied or highly satisfied (eFigures 1 and 2 in the Supplement).

The primary objective of shifting more than 50% of ambulatory clinic visits to VC was achieved 4 days after VCMS deployment (249 VC visits vs 239 in-person visits). During the study period, 22 085 VC visits (17 888 telephone visits and 4197 video visits) occurred, which equates to a median of 69.0% (range, 18.8%-100%) of daily clinic visits compared with 1.38% (0-9 visits per day) during the prior month (Figure 1). After the pandemic declaration, ambulatory visits decreased by a mean (SD) of 24.9% (2.4%) (*P* < .001) (eFigure 3 in the Supplement). Outpatient clinic case volumes were restored within a month (estimate for April 20 to May 22, −0.03; 95% CI, −0.10 to 0.03; *P* = .36) (eTable 2 in the Supplement), a recovery not observed at other tertiary hospitals nearby (eTable 3 in the Supplement). Data on chemotherapy and radiotherapy visits and quality indexes for safety and timeliness of oncologic care are given in eFigures 4 and 5 in the Supplement.

**Figure 2.**
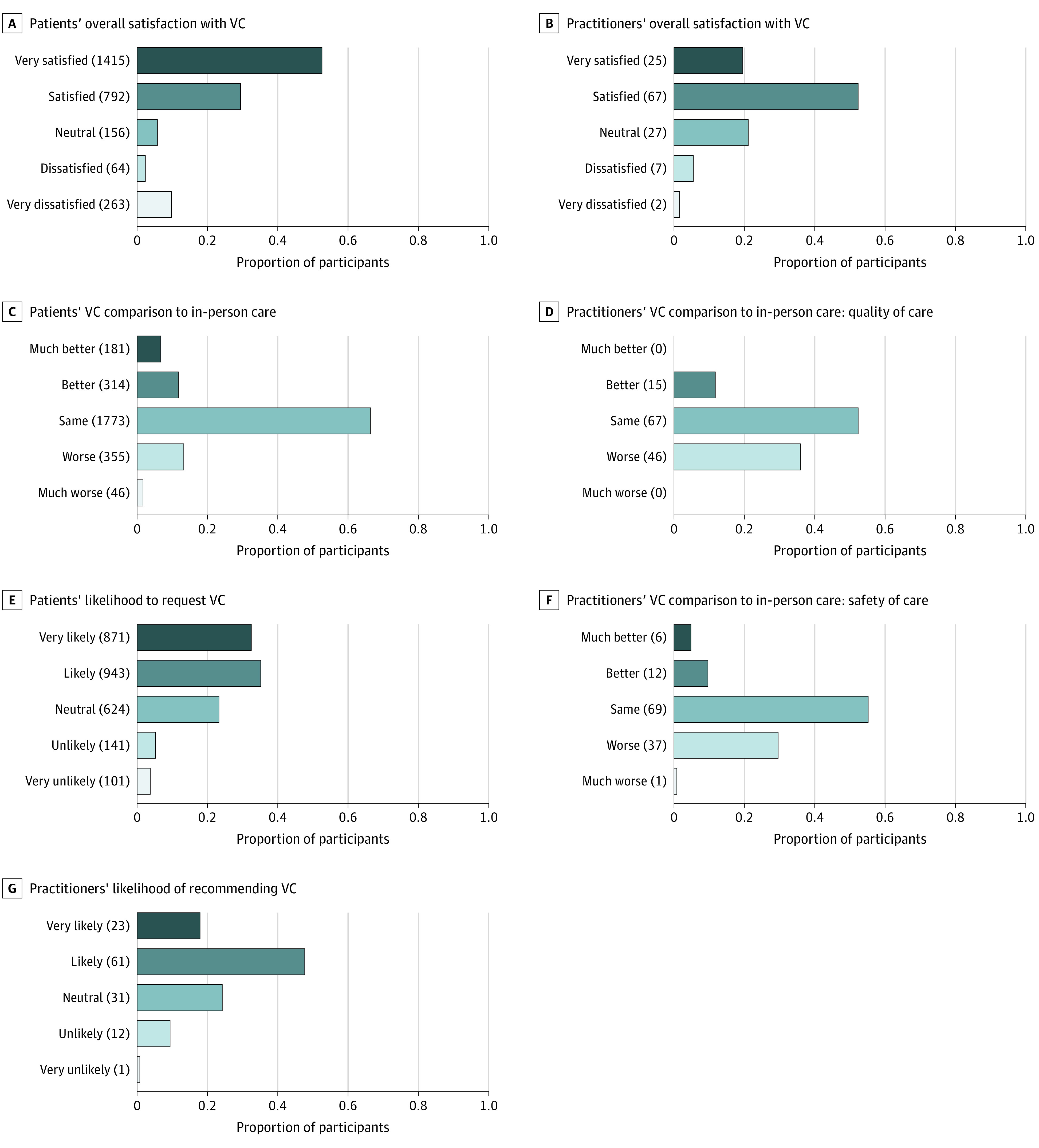
Patient and Practitioner Satisfaction Survey Individual Responses VC indicates virtual care.

A total of 3507 (24% response rate) patient satisfaction survey responses were received; of these, 2738 corresponded to a single individual with a registered Ontario postal code and were included in the analyses (eTables 4 and 5 in the Supplement). Patient experience survey results are shown in Figure 2. Overall, patients were highly satisfied with VC (1808 [68%] recommended this care model), independent of VC modality. Those undergoing video calls were more likely to consider them better than in-person visits (105 [24%] vs 385 [17%] [telephone]; *P* = .006) and to request VC for their future appointments (330 [77%] vs 1478 [66%] [telephone]; *P* < .001) (eTables 6 and 7 in the Supplement). Multivariable models showed that overall patient satisfaction was associated with sex and income but not with VC modality, age, or inequality indexes. The ethnocultural composition index (self-identification as visible minority, foreign born, linguistic isolation, and recent immigration) (eMethods in the Supplement) was associated with a favorable rating of VC but, paradoxically, a lower likelihood of requesting it for future appointments (eTable 8 in the Supplement). Overall practitioner satisfaction was comparable to that of patients (Figure 2; eFigures 6 and 7 and eTables 9 through 11 in the Supplement). However, a higher proportion of physicians thought that VC led to compromises in patient care compared with in-person visits (46 [36%] in quality and 38 [31%] in safety of care vs 401 [15%] of patients for overall comparison); these perceptions improved over time (eTable 12 in the Supplement).

Of the total VC visits, travel data were retrievable for 21 204 cases (96%) cases, and 19 505 patients (88%) had an Ontario address registered that could be mapped to census data. Displacement-related savings per patient are given in the Table, translating in overall cost savings of CAD$ 3 014 602 to CAD$ 3 155 946 for 22 085 patients during the study period.

**Table.** Estimated Per-Patient Cost Savings With the Shift to Virtual Care in Displacement-Related Burden During the Study Period

Variable	Transportation, median (IQR)	
	Public	Private
Travel		
1-Way travel distance, km	17.6 (8.6-37.3)	21.5 (8.2-42.5)
2-Way travel time, min	58.9 (40.5-88.8)	29.3 (19.7-40.8)
2-Way travel expense plus parking (if applicable), CAD$	6.5 (6.5-6.5)	42.8 (28.7-65.1)
Cost, CAD$		
2-Way opportunity cost plus time on premises	130 (95.1-188)	99.2 (72-133.6)
Total travel and opportunity cost	136.5 (98-194.5)	142.9 (107.2-199.2)

Abbreviation: IQR, interquartile range.

## Discussion

The findings of this cohort study support the use of VC for optimizing outpatient cancer care during and after the COVID-19 pandemic. The study of VC in oncology has been limited to medium-sized case series, which explored customized digital interventions for specific diseases, clinical scenarios, or symptoms.^8^ Some specialties have reported on their VC initiatives in response to COVID-19, but residual negative associations with service volumes and a lack of multidomain quality data remain prevalent.^9,10,11^ Comparably high-volume tertiary cancer centers in North America^12^ and Asia^13^ and a large network of practitioners in the US^14^ have increasingly adopted VC (approximately 15%-60%); however, paired with a gross reduction of outpatient volumes, concerns regarding COVID-19 and its impact on the care of patients with cancer have surfaced. These issues highlight the relevance of the current study’s service design approach, including the streamlining of services for developing the VCMS, because it enabled the restoration of pre–COVID-19 case volumes without measurable compromise on quality domains, thus allowing in-person visits to be prioritized for patients requiring treatments.

Several factors contributed to the rapid implementation of this initiative across the PM. First was the cohesive support across all stakeholders to meet a clearly communicated organizational goal. Second, our discovery work steered us toward developing a digital wrapper solution to amalgamate existing processes and tools, thus maximizing speed and minimizing disruption. Third, new VC fee codes were provincially approved at an early stage, aligning the practitioners’ compensation to that of in-person care. Nonetheless, adequate reimbursement seems necessary but itself insufficient to fuel the widespread adoption of VC.^12,13,14^

### Limitations

This study has limitations. First, because of the short duration of follow-up, this study did not include data on oncologic outcomes of patients receiving VC. Existing literature suggests that VC may not compromise disease-specific outcomes.^15^ Long-term data will provide information on this important clinical end point and the sustainability of VC models. Second, the study was from a single institution; thus, the described process and methods may require adaptation for other clinical settings.

## Conclusions

This work provides quantitative data to characterize the value proposition of oncologic VC at scale. Nonetheless, additional work is required to delineate the optimal integration and modalities of VC visits. Progressive financing mechanisms, regulatory and data security frameworks with bespoke legislations, digital literacy of patients and practitioners, and integration of multidisciplinary care teams will be paramount to allow patients to access modern and high-quality care from their homes.

## Notes

Supplement. ** eMethods. **Supplementary Methods **eFigure 1.** Uptake and Use of VCMS **eFigure 2.** User Satisfaction With VCMS **eFigure 3.** Total Number of Weekly Ambulatory Visits Over Time **eFigure 4.** Outpatient Volumes for Cancer Treatments Requiring In-person Visits **eFigure 5.** Regularly Monitored Safety and Timeliness Indicators Over Time **eFigure 6.** Physician Satisfaction Survey Results Stratified by Provider Type **eFigure 7.** Physician Satisfaction Survey Results Stratified by Communication Type **eTable 1.** Project Direct Costs **eTable 2.** Multivariable Log-Linear Model of Ambulatory Visits at PM Over Time **eTable 3.** Multivariable Log-Linear Model of Ambulatory Visits Over Time at Two Comparable Tertiary-Hospitals **eTable 4.** Survey Completion Rates **eTable 5.** Summary of Patient Characteristics **eTable 6.** Patient Satisfaction Stratified by VC Modality **eTable 7.** Complete Survey Responses: Patients **eTable 8.** Multivariable Analyses on the Patient-Reported Satisfaction With VC **eTable 9.** Complete Survey Responses: Physicians **eTable 10.** Complete Survey Responses: Administrative Assistants **eTable 11.** Complete Survey Responses: Patient Flow Coordinator **eTable 12.** Logistic Regression of Provider’s Responses Over Time **eReferences**
