# Supplementary material for: Implementation and Outcomes of Virtual Care Across a Tertiary Cancer Center During COVID-19
Source: JAMA Oncol. 2021 Jan 7;7(4):1–6. doi: 10.1001/jamaoncol.2020.6982 (PMC7791400; doi:10.1001/jamaoncol.2020.6982)
Supplement: Supplement. — eMethods. Supplementary Methods eFigure 1. Uptake and Use of VCMS eFigure 2. User Satisfaction With VCMS eFigure 3. Total Number of Weekly Ambulatory Visits Over Time eFigure 4. Outpatient Volumes for Cancer Treatments Requiring In-person Visits eFigure 5. Regularly Monitored Safety and Timeliness Indicators Over Time eFigure 6. Physician Satisfaction Survey Results Stratified by Provider Type eFigure 7. Physician Satisfaction Survey Results Stratified by Communication Type eTable 1. Project Direct Costs eTable 2. Multivariable Log-Linear Model of Ambulatory Visits at PM Over Time eTable 3. Multivariable Log-Linear Model of Ambulatory Visits Over Time at Two Comparable Tertiary-Hospitals eTable 4. Survey Completion Rates eTable 5. Summary of Patient Characteristics eTable 6. Patient Satisfaction Stratified by VC Modality eTable 7. Complete Survey Responses: Patients eTable 8. Multivariable Analyses on the Patient-Reported Satisfaction With VC eTable 9. Complete Survey Responses: Physicians eTable 10. Complete Survey Responses: Administrative Assistants eTable 11. Complete Survey Responses: Patient Flow Coordinator eTable 12. Logistic Regression of Provider’s Responses Over Time eReferences [file jamaoncol-e206982-s001.pdf]

# Supplementary Online Content

Berlin A, Lovas M, Truong T, et al. Implementation and outcomes of virtual care across a tertiary cancer center during COVID-19. *JAMA Oncol.* Published online January 7, 2021.
doi:10.1001/jamaoncol.2020.6982

**eMethods.** Supplementary Methods
**eFigure 1.** Uptake and Use of VCMS
**eFigure 2.** User Satisfaction With VCMS
**eFigure 3.** Total Number of Weekly Ambulatory Visits Over Time
**eFigure 4.** Outpatient Volumes for Cancer Treatments Requiring In-person Visits
**eFigure 5.** Regularly Monitored Safety and Timeliness Indicators Over Time
**eFigure 6.** Physician Satisfaction Survey Results Stratified by Provider Type
**eFigure 7.** Physician Satisfaction Survey Results Stratified by Communication Type
**eTable 1.** Project Direct Costs
**eTable 2.** Multivariable Log-Linear Model of Ambulatory Visits at PM Over Time
**eTable 3.** Multivariable Log-Linear Model of Ambulatory Visits Over Time at Two Comparable Tertiary-Hospitals
**eTable 4.** Survey Completion Rates
**eTable 5.** Summary of Patient Characteristics
**eTable 6.** Patient Satisfaction Stratified by VC Modality
**eTable 7.** Complete Survey Responses: Patients
**eTable 8.** Multivariable Analyses on the Patient-Reported Satisfaction With VC
**eTable 9.** Complete Survey Responses: Physicians
**eTable 10.** Complete Survey Responses: Administrative Assistants
**eTable 11.** Complete Survey Responses: Patient Flow Coordinator
**eTable 12.** Logistic Regression of Provider's Responses Over Time
**eReferences**

**This supplementary material has been provided by the authors to give readers additional information about their work.**

© 2020 Berlin A et al. *JAMA Oncology.*

**eMethods.** Supplementary Methods

## Materials and Methods

Agile Service Design processes were employed to rapidly understand the current state of the clinics at PM, ideate a near future state that would allow for the mass-redistribution of appropriate patients from in-person into virtual care. Service Design has been described as a mindset, a set of methods, and a process to create a new service in a human-centered way[1]. To execute this endeavour, a core project team of three was formed from the Smart Cancer Care Program (PM, UHN), including: medical lead, information scientist, and a service designer/engineer. Additionally, a close partnership with an institutional technology team (Health Informatics Research [HIR], Techna Institute, UHN) was established. Likewise, the endeavour translated in direct involvement of clinical champions, hospital administration, and representation from patients. The overall initiative process included distinct phases of discovery, ideation, prototype and testing, launch, data collection and continuous improvement:

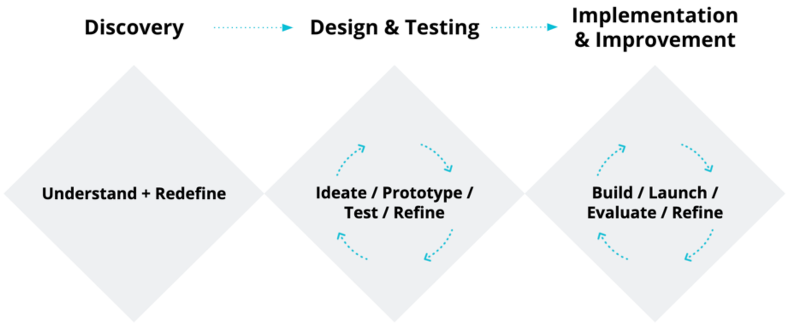


Framework for innovation and its distinct phases. *Adapted from Eisermann R et al.* [2]

### Discovery

Typical Service Design discovery borrows from ethnographic-style research methods in which a carefully selected sample of each user population is engaged, sessions are deeply considered and rehearsed to maximize the depth of the understanding that is generated from each activity[3]. With the extreme time pressures, our discovery relied on the rapid selection of front-line staff who were available during a 2-day blitz. We conducted many short, semi-structured interviews to collect information about existing workflows, communications, and moments of struggle for staff and patients. Additionally, we flagged any processes that involved verbal communication, paper forms, email, or spreadsheets as candidates for redesign. We also collected physical artifacts, such as physician order forms during the blitz. Priority was placed on obtaining information from first-hand accounts of the front-line users (eg: patient flow coordinators, nurses, admins, and physicians). Aligned with our primary goal, we focused our

© 2020 Berlin A et al. *JAMA Oncology.*

efforts in the 80% of clinics with highest patient volumes clinics and on workflows associated with patients that would be most amenable to virtual care, such as well-follow-up visits.

**Prior to Clinic Workflows**

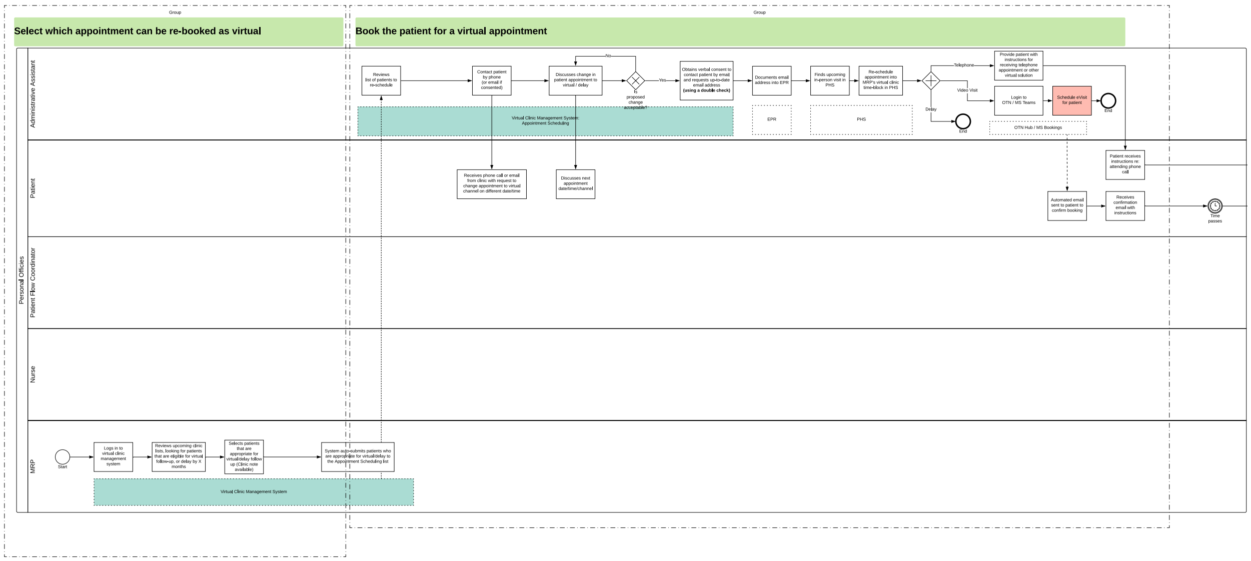


**Virtual Clinic Workflows**

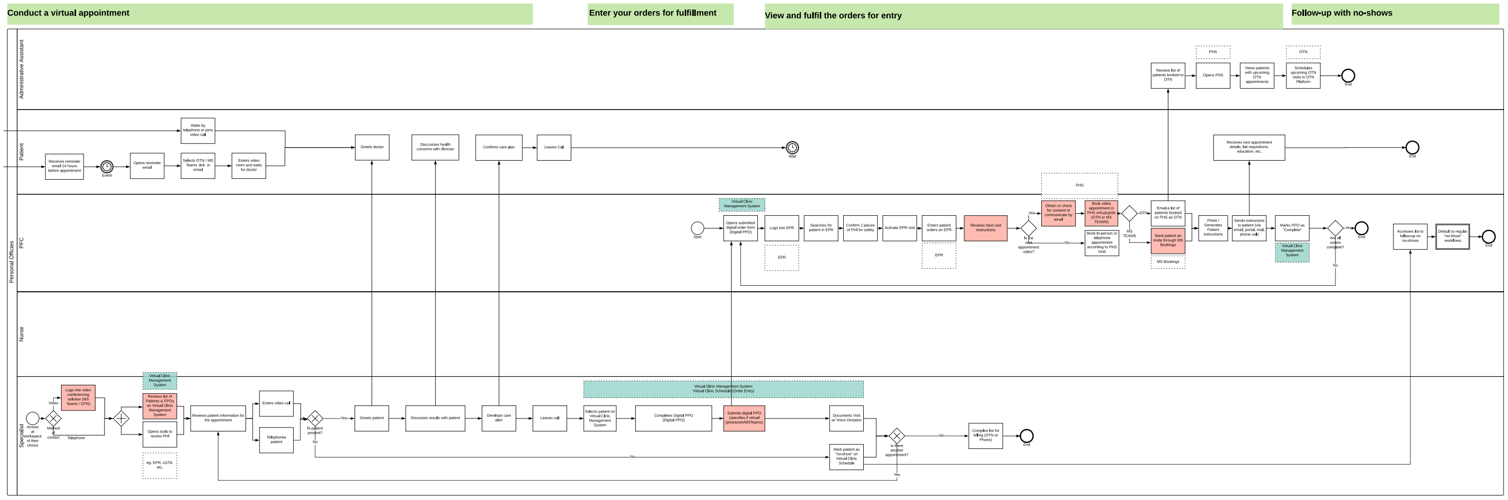


*Ambulatory clinic service mapping. Includes, prior (top) and during (bottom) VC visit workflow. Tale blue bars represent mapped points and transitions for which a digital solution was considered required to help shifting towards VC at scale. Figure can be accessed at:* https://app.lucidchart.com/invitations/accept/43e837f1-34aa-40e0-9783-20f7ae7e59f2

© 2020 Berlin A et al. *JAMA Oncology.*

Ideation

Once workflows were mapped and vetted with relevant managers to ensure the broad applicability across the cancer centre, the team began ideating solutions to overcoming the known and anticipated challenges with healthcare teams being remote and dislocated. Two major hurdles emerged out of the discovery and ideation phases: i) physicians would be required to triage hundreds of patients for upcoming in-person visits —safely deciding whom were candidates for virtual care— and then clearly communicate this to the administrative assistants for re-booking, and ii) care teams traditionally relied on paper-based process of capturing and communicating physician orders to administrative staff and nurses, which would not be safe, practical, or timely for communications between remote staff. The focus of ideation sessions conducted over video calls centered on solutions of these two challenges, while a backlog of other challenging areas and ideas were captured for future developments.

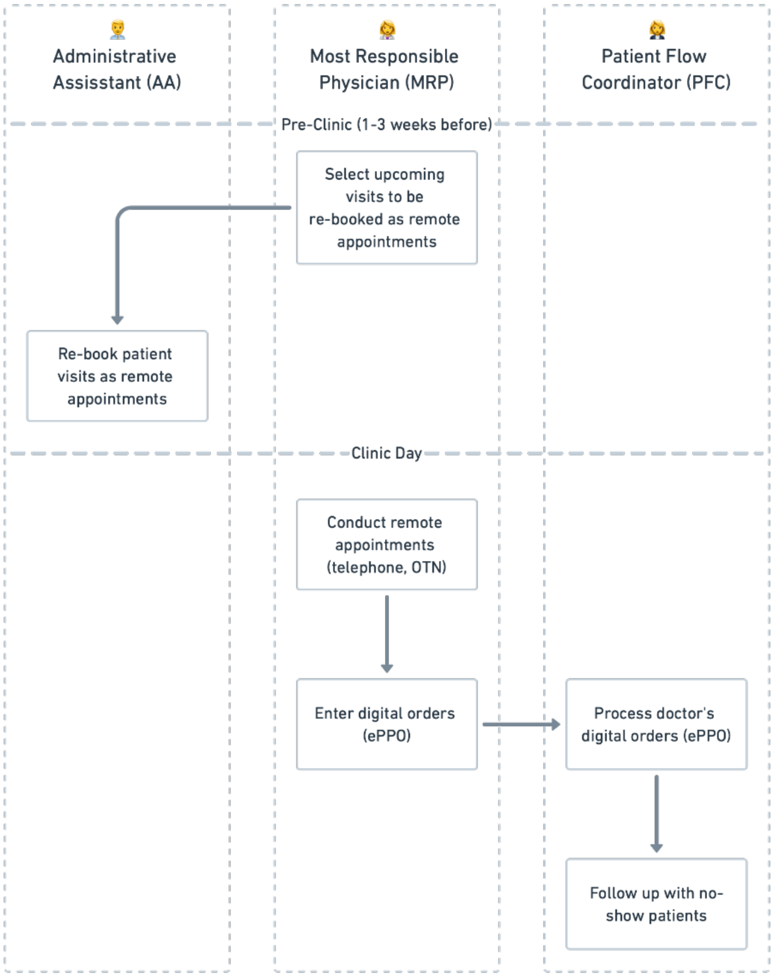


*Simplified workflow diagram for the ambulatory clinic VC service envisioned at time of the Virtual Care Management System (VCMS) ideation process. OTN: Ontario Telehealth Network (Ministry of Health-supported video call solution for patient-provider interactions in Ontario)*

© 2020 Berlin A et al. *JAMA Oncology.*

Prototyping and Testing

A hallmark of human centered design is the rapid creation of prototypes, which are then to be tested with real end-users in order to facilitate rapid iterations[4]. In Service Design, prototypes take many forms, from new processes, to multimedia training materials, websites, to digital tools. For the challenges at-hand, in collaboration with designers at Healthcare Human Factors (UHN), we prototyped new processes (Lucid Software Inc., US) and new digital tools (Figma Inc., US) to rapidly test the prototypes with key stakeholders – physicians and administrative assistants for the triage solution, and physicians and patient flow coordinators for the ordering solution. Feedback was gathered and folded back into new iterations with vetting from the team. This included discernment from strong clinical champions, who were well versed in technology, design, and clinical practice to separate feedback that was an outlier in the clinical landscape, as opposed to the norm. Technical representatives and clinical managers were also involved throughout this phase to ensure that the technical and clinical implications were feasible within the aggressive timelines. Development and quality assurance were executed by the HIR technology team, and then uploaded to internal servers.

Launch

Secure remote access was enabled (Citrix Receiver, Citrix Systems Inc, US) for all relevant users. Working closely with clinical champions, clinic managers and directors, training was conducted for all users over the weekend before launch (total 10 virtual sessions). Additionally, materials describing the workflows and VCMS functionalities were made available online (https://www.notion.so/PM-Virtual-Clinic-Guides-3728eae6638541b3b7287ecd9b469c92). Super-users were self-identified after this training and were provided further training. Subsequently, these super-users offered informal peer-to-peer training and support. In collaboration with our Institutional IT team, UHN Digital, three-tier support was offered to all users through the enterprise-wide ticketing and tech-support system (ServiceNow Inc., US). High volume and high impact tickets from the support system are tagged and thematically analyzed as they arrive. Bugs are fixed immediately, while the other tickets are prioritized to be addressed in our 2-week development sprints.

Data Collection and Continuous Improvement

Data collection was critical to the on-going improvement of the system after launch. Clinic volumes and visit types over time were federated daily from the existing scheduling system (PHS, McKesson, US) using a bespoke data analytics instance (Power BI, Microsoft Corporation, US) created for monitoring the impact of this initiative.

Assessment indices

The impact of this initiative was evaluated across three domains: cancer care delivery, patients' and providers' experiences with VC and potential cost-savings; we assessed elements from the six domains of quality outlined by the Institute of Medicine: effectiveness, safety, timeliness, patient-centredness, equitability and efficiency[5]. Considering COVID-19 consequences on multiple dimensions of healthcare beyond cancer care, attributable effectiveness was measured through the outpatient volumes (both ambulatory clinic and ambulatory chemotherapy and radiotherapy visits) over time.  Institution- and provincial-based quality

© 2020 Berlin A et al. *JAMA Oncology.*

indicators were analyzed to evaluate safety (incident reports, Hepatitis B screening before chemotherapy start, discharge summary completion) and timeliness (time from referral to consultation). Patients and providers' experiences with VC were captured using voluntary and confidential surveys (SoGoSurvey, US; see pages 7-14). Daily invitations were sent via email or SMS to patients, and e-mail invitations to providers (physicians, AAs, PFCs) after week 1 and 6 following VCMS deployment. Associations between patient-reported satisfaction and inequality indices were assessed by matching postal codes to dissemination area (DA)-level socio-demographic statistics capturing the four dimensions of the Canadian index of multiple deprivation[6] (CIMD, *see page* 15). Patients' income was estimated based on their neighborhood average from the Statistics Canada 2016 census data (Postal Code Conversion File [PCCF+], version 7B). Displacement-related cost-savings were estimated using Google distance matrix API (Google LLC, US) from postal code to PM. Retrieved travel times at 0700, 1200 and 1600 hrs were averaged for each individual. Toronto Transit Commission fare ($3.25CAD) for each segment and the standard automobile allowance rate (Canada Revenue Agency 2020, $0.53CAD/Km) were applied for public and private transportation, respectively. For the latter, $20CAD was added for parking costs, based on current rates. Opportunity cost was calculated by applying the average income by postal code and average working-hours/week for the corresponding age-group to the travel time for each individual and two-hours of non-clinical encounter time on-premises during in-person visits.

© 2020 Berlin A et al. *JAMA Oncology.*

## Patient Satisfaction Survey

At Princess Margaret, we are always striving to improve the patient experience. You are receiving this survey because you had an appointment in the last week with your doctor over the phone or by video. These types of appointments are referred to as "virtual care" or "remote appointments".

Your contribution is key in understanding how we can improve virtual care. Your participation is voluntary, and all answers will be kept confidential. If you decide not to complete this survey, your care at UHN will not be affected.

**Please answer the following questions about your recent remote appointment:**

**1. What form of communication was used for your recent remote appointment? (Select one option)**

○ Phone call

○ Video call

**2. Overall, how satisfied were you with your remote appointment?**

| | Very Dissatisfied | Dissatisfied | Neutral | Satisfied | Very Satisfied |
|---|---|---|---|---|---|
| (a) Satisfaction | ○ | ○ | ○ | ○ | ○ |

**3. How did your remote appointment compare to your usual in-person visit?**

| | Much Worse | Worse | Same | Better | Much Better |
|---|---|---|---|---|---|
| (a) Comparison | ○ | ○ | ○ | ○ | ○ |

**4. How likely would you be to request another remote appointment?**

| | Very Unlikely | Unlikely | Neutral | Likely | Very Likely |
|---|---|---|---|---|---|

© 2020 Berlin A et al. *JAMA Oncology.*

(a) Likelihood ○ ○ ○ ○ ○

**NOTE :** Answer the below question only if answer to Q#4(a) is Very Unlikely OR Unlikely OR Neutral OR Likely OR Very Likely

**Why? Because of...**
**(select all that apply)**

- ☐ Form of communication (phone/video)
- ☐ Performance of technology
- ☐ Quality of care
- ☐ Convenience
- ☐ Punctuality
- ☐ Other (Please specify) _______________

**5. Do you have any suggestions to help us improve virtual care at Princess Margaret?**
(free text, limit 560 characters)

© 2020 Berlin A et al. *JAMA Oncology.*

UHN Princess Margaret Cancer Centre — smart cancer care

# Physician Satisfaction Survey

**Smart Cancer Care is striving to improve workflow efficiency and user experience in transitioning to virtual care—your contribution is key. Your participation is voluntary, and all answers will be kept confidential.**

**1. Over the past week, what form of communication have you used for most of your remote appointments? (Select one option)**

- ○ Phone call
- ○ Video call

**2. Over the past week, how satisfied have you been with your remote appointments?**

| | Very Dissatisfied | Dissatisfied | Neutral | Satisfied | Very Satisfied |
|---|---|---|---|---|---|
| (a) Satisfaction | ○ | ○ | ○ | ○ | ○ |

**3. Overall, what is the impact of remote appointments on your delivery of care?**

| | Much Worse | Worse | Same | Better | Much Better |
|---|---|---|---|---|---|
| (a) Quality of Care | ○ | ○ | ○ | ○ | ○ |
| (b) Safety of Care | ○ | ○ | ○ | ○ | ○ |
| (c) Work Efficiency | ○ | ○ | ○ | ○ | ○ |

**4. How likely are you to recommend conducting remote appointments to clinicians who see similar patients to you?**

| | Very Unlikely | Unlikely | Neutral | Likely | Very Likely |
|---|---|---|---|---|---|
| (a) Likelihood | ○ | ○ | ○ | ○ | ○ |

© 2020 Berlin A et al. *JAMA Oncology.*

**NOTE :** Answer the below question only if answer to Q#4(a) is Very Unlikely OR Unlikely OR Neutral OR Likely OR Very Likely

**Why? Because of...**
**(select all that apply)**

- ☐ Form of communication (phone/video)
- ☐ Performance of technology
- ☐ Quality of care
- ☐ Safety of care
- ☐ Efficiency
- ☐ Convenience
- ☐ Other (Please specify) _______________

---

**5. Over the past week, how satisfied have you been with the Virtual Care Management System (VCMS) for assisting you with managing your remote appointments?**

| | Very Dissatisfied | Dissatisfied | Neutral | Satisfied | Very Satisfied | Not Applicable |
|---|---|---|---|---|---|---|
| (a) Satisfaction | ○ | ○ | ○ | ○ | ○ | ○ |

**6. Do you have any suggestions to help us improve virtual care at Princess Margaret?**
(free text, limit 560 characters)

© 2020 Berlin A et al. *JAMA Oncology.*

## Administrative Assistant Satisfaction Survey

Smart Cancer Care is striving to improve workflow efficiency and user experience in transitioning to virtual care—your contribution is key. Your participation is voluntary, and all answers will be kept confidential.

If you are not using the Virtual Care Management System (VCMS) yet, please select "Not Applicable" where applicable.

**1. Over the past week, how satisfied have you been with the VCMS for assisting you in rebooking appointments?**

| | Very Dissatisfied | Dissatisfied | Neutral | Satisfied | Very Satisfied | Not Applicable |
|---|---|---|---|---|---|---|
| (a) Satisfaction | ○ | ○ | ○ | ○ | ○ | ○ |

**2. Overall, what is the impact of the VCMS on:**

| | Much Worse | Worse | Same | Better | Much Better | Not Applicable |
|---|---|---|---|---|---|---|
| (a) Work Efficiency | ○ | ○ | ○ | ○ | ○ | ○ |
| (b) Booking Errors | ○ | ○ | ○ | ○ | ○ | ○ |
| (c) Communication with Physician(s) | ○ | ○ | ○ | ○ | ○ | ○ |

**3. Over the past week, how has working remotely compared with working at the hospital?**

| | Much Worse | Worse | Same | Better | Much Better | I'm not working remotely |
|---|---|---|---|---|---|---|
| (a) Comparison | ○ | ○ | ○ | ○ | ○ | ○ |

**4. How likely are you to recommend using the VCMS to manage virtual clinics?**

© 2020 Berlin A et al. *JAMA Oncology.*

| | Very Unlikely | Unlikely | Neutral | Likely | Very Likely | Not Applicable |
|---|---|---|---|---|---|---|
| (a) Likelihood | ○ | ○ | ○ | ○ | ○ | ○ |

**NOTE :** Answer the below question only if answer to Q#4(a) is Very Unlikely OR Unlikely OR Neutral OR Likely OR Very Likely

**Why? Because of...**
**(select all that apply)**

- ☐ Performance of technology
- ☐ Efficiency
- ☐ Communication with physician
- ☐ Convenience
- ☐ Other (Please specify) _______________

**5. Do you have any suggestions to help us improve virtual care at Princess Margaret?**
(free text, limit 560 characters)

© 2020 Berlin A et al. *JAMA Oncology.*

## Patient Flow Coordinator Satisfaction Survey

Smart Cancer Care is striving to improve workflow efficiency and user experience in transitioning to virtual care—your contribution is key. Your participation is voluntary, and all answers will be kept confidential.

If you are not using the Virtual Care Management System (VCMS) yet, please select "Not Applicable" where applicable.

**1. Over the past week, how satisfied have you been with VCMS for assisting you in completing physician orders?**

| | Very Dissatisfied | Dissatisfied | Neutral | Satisfied | Very Satisfied | Not Applicable |
|---|---|---|---|---|---|---|
| (a) Satisfaction | | | | | | |

**2. Overall, what is the impact of the VCMS on:**

| | Much Worse | Worse | Same | Better | Much Better | Not Applicable |
|---|---|---|---|---|---|---|
| (a) Work Efficiency | | | | | | |
| (b) Clarity and Completeness of Orders (eg. ePPO) | | | | | | |
| (c) Communication with Care Team | | | | | | |

**3. Over the past week, how has working remotely compared with working at the hospital?**

| | Much Worse | Worse | Same | Better | Much Better | I'm not working remotely |
|---|---|---|---|---|---|---|
| (a) Comparison | | | | | | |

© 2020 Berlin A et al. *JAMA Oncology.*

**4. How likely are you to recommend using the VCMS to process physician orders?**

| | Very Unlikely | Unlikely | Neutral | Likely | Very Likely | Not Applicable |
|---|---|---|---|---|---|---|
| (a) Likelihood | ○ | ○ | ○ | ○ | ○ | ○ |

**NOTE :** Answer the below question only if answer to Q#4(a) is Very Unlikely OR Unlikely OR Neutral OR Likely OR Very Likely

**Why? Because of...**
**(select all that apply)**

- ☐ Performance of technology
- ☐ Efficiency
- ☐ Communication with care team
- ☐ Digital orders (ePPO)
- ☐ Convenience
- ☐ Other (Please specify) _______________

**5. Do you have any suggestions to help us improve virtual care at Princess Margaret?** (free text, limit 560 characters)

© 2020 Berlin A et al. *JAMA Oncology.*

Canadian index of Multiple deprivation

The four dimensions of multiple deprivation and their corresponding indicators, Canada, 2016[6].

1) ***Residential instability***, includes the following indicators:
    i. proportion of dwellings that are apartment buildings,
    ii. proportion of dwellings that are owned[#],
    iii. proportion of persons living alone,
    iv. proportion of the population who moved within the past five years,
    v. proportion of population that is married or common-law[#].
2) ***Economic dependency***, includes the following indicators:
    i. proportion of population aged 65 and older,
    ii. proportion of population participating in labour force (aged 15 and older)[#],
    iii. ratio of employment to population[#],
    iv. dependency ratio (population aged 0-14 and aged 65 and older divided by population aged 15-64),
    v. proportion of population receiving government transfer payments.
3) ***Ethno-cultural composition***, includes the following indicators:
    i. proportion of population who self-identify as visible minority,
    ii. proportion of population that is foreign-born,
    iii. proportion of population with no knowledge of either official language (linguistic isolation),
    iv. proportion of population who are recent immigrants (arrived in five years prior to Census).
4) ***Situational vulnerability***, includes the following indicators:
    i. proportion of population that identifies as Aboriginal,
    ii. proportion of dwellings needing major repairs,
    iii. proportion of population aged 25-64 without a high school diploma.

Note: The dimensions are ordered such that the first dimension explains the highest percentage of the variance of the data and the last dimension explains the lowest percentage.
[#] This indicator was reverse-coded, meaning it was coded opposite of the measure. For example, proportion of population that is married or common-law becomes proportion of population that is single, divorced, separated or widowed.

© 2020 Berlin A et al. *JAMA Oncology.*

Statistical Analyses

Data were summarized using descriptive statistics, including frequencies and proportions for categorical variables. Continuous variables were summarized using means with standard deviation (SD) or medians with range or interquartile range (IQR). Chi-square test was used for comparison of survey responses between VC modalities.

To assess association between sociodemographic factors and VC modality with patient survey response, multivariable ordinal logistic regression was used. Univariable ordinal logistic regression was carried out to compare responses between the two rounds of surveys among providers. Observations with missing data in either responses or demographics were excluded. To account for intra-participant correlation due to multiple surveys completed by the same individual, clustered robust standard errors were used to calculate Wald test p-values. Brant test was used to assess proportionality assumption in the model.

To assess if the volume of ambulatory visits post-VCMS deployment were restored to pre-COVID-19 levels, multivariable log-linear models were fitted to the daily number of visits (on log scale), adjusting for the number of visits in the previous day (on log scale) to account for the correlation over time. Weekends and statutory holidays were excluded due to low numbers.

To compare quality of care between pre-COVID and COVID period, multivariable linear models were fitted to each of four monthly reported safety and timeliness indicators adjusting for the performance in the previous month (i.e. lagged proportion or count). These indicators include (i) number of incident reports in the Radiation Medicine Program, (ii) proportion of patients who had Hepatitis B virus screening before first systemic therapy start, (iii) proportion of discharge summaries completed within 48 hours, (iv) proportion of referrals that were seen within 14 days.

All tests were two-sided, and a threshold of $P < 0.05$ was set for statistical significance. All analyses were performed in the R statistical environment (v3.5.2), and available upon request.

© 2020 Berlin A et al. *JAMA Oncology.*

## Supplementary Figures

**eFigure 1.** Uptake and use of VCMS over the study period time (March 23 to May 22, 2020). In total, 431 users had active accounts, corresponding to 239 physicians, 52 nurses, 87 administrative assistants and 53 patient flow coordinators (representing 67%, 48%, 64% and 76% of registered users, respectively). In average, 47 digital orders were submitted during the first week post VCMS deployment (representing 14% [3-28%] of the VC activity); increasing to 404 orders during the last week of this study (representing 69% [57-73%] of the VC activity).

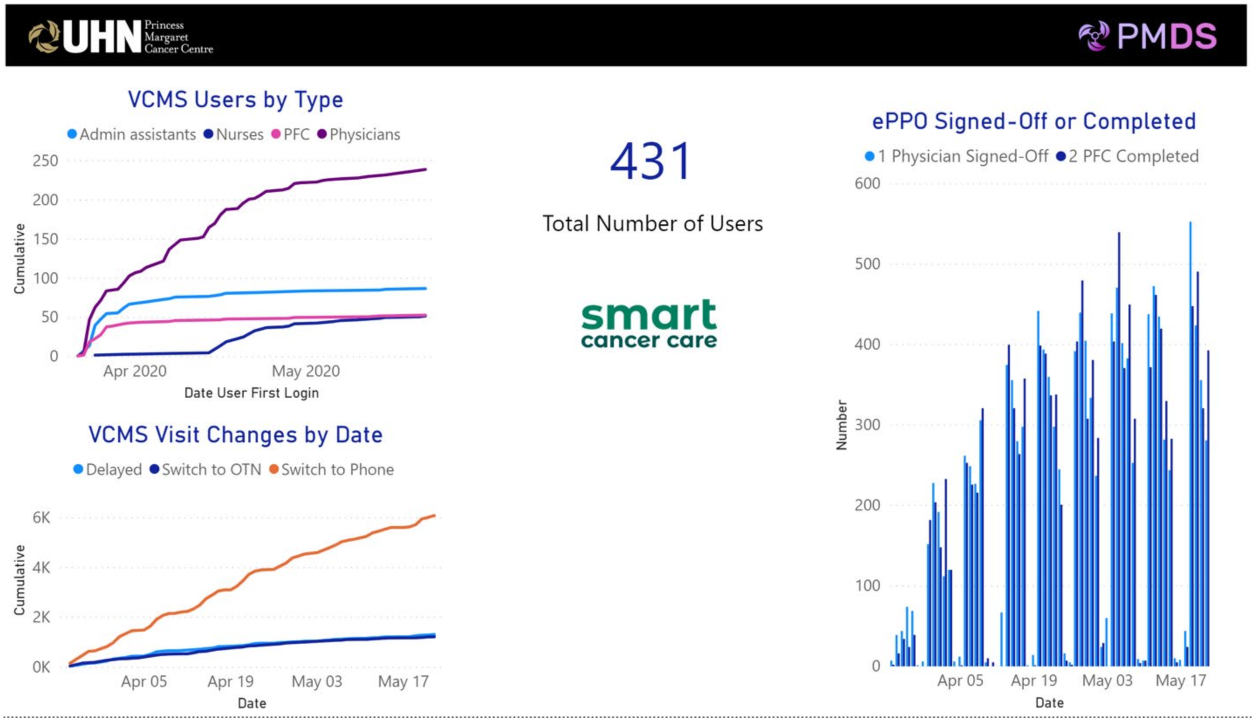


© 2020 Berlin A et al. *JAMA Oncology.*

**eFigure 2**. User satisfaction with VCMS, and likelihood to recommend it for managing VC. AAs: administrative assistants; PFCs: patient flow coordinators

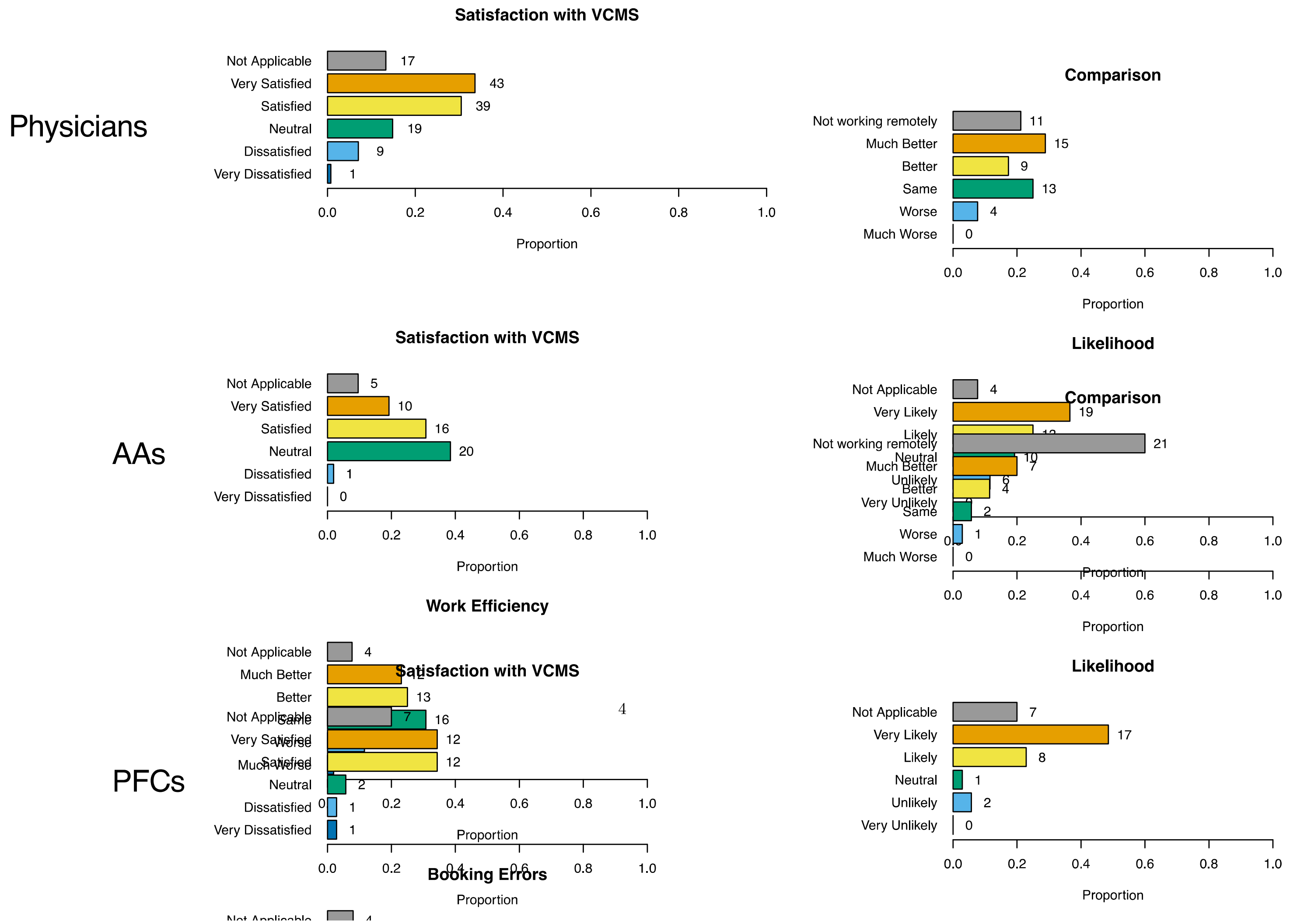


© 2020 Berlin A et al. *JAMA Oncology.*

**eFigure 3.** Total number of weekly outpatient clinic visits over time from February 17 (week 8) to May 22 (week 21), 2020.

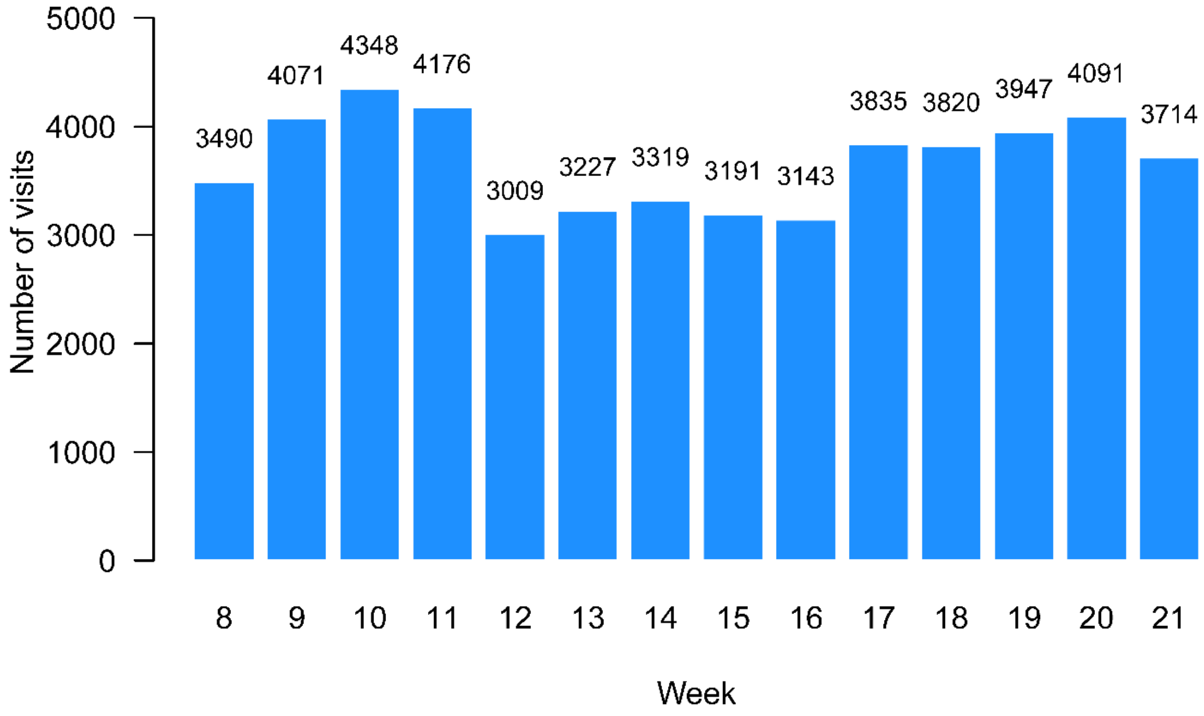


© 2020 Berlin A et al. *JAMA Oncology.*

**eFigure 4.** Outpatient volumes for cancer care treatments requiring in-person visits. Total weekly patient visits for the delivery of chemotherapy and radiation treatments, from February 17 (week 8) to May 22 (week 21), 2020.

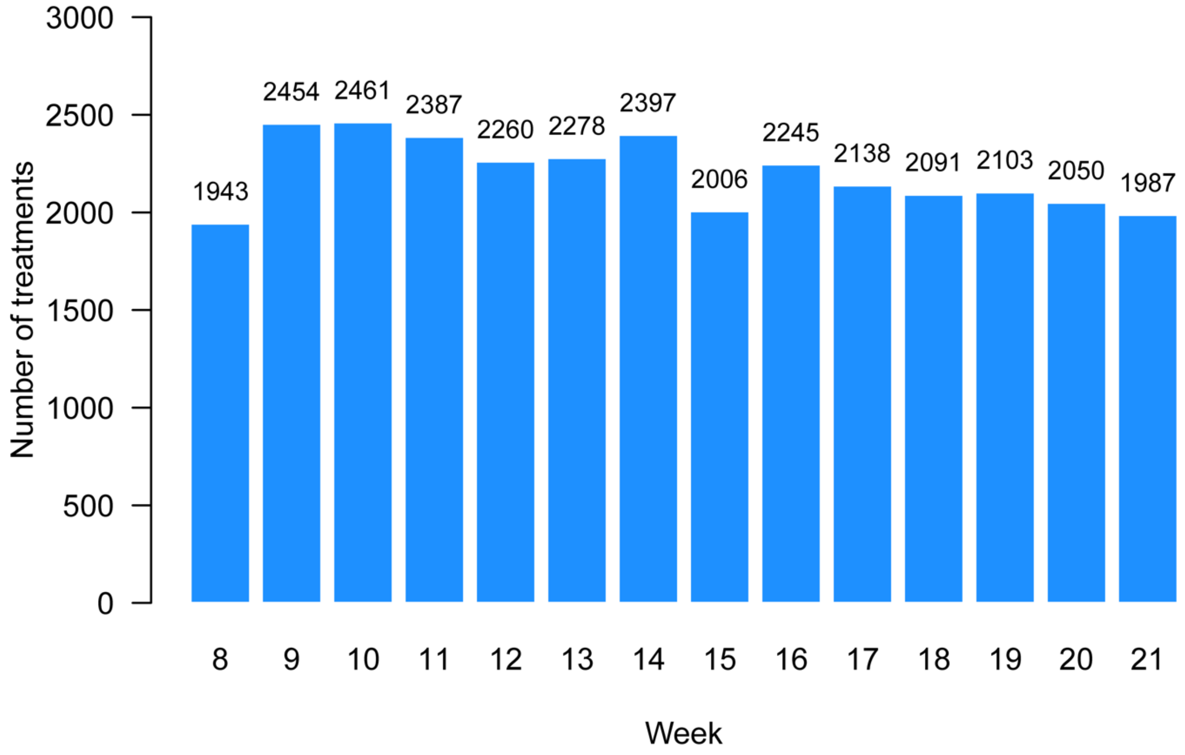


© 2020 Berlin A et al. *JAMA Oncology.*

**eFigure 5.** Regularly monitored safety and timeliness indicators over time. To compare quality of care between pre-COVID period (before March 2020) and COVID period (March to May 2020), four indicators were examined: A) total number of incident reports in the Radiation Medicine Program, B) Hepatitis B virus (HBV) screening before first systemic therapy start, C) discharge summary completion rates (within 48 hours), D) time from referral to consult (within 14 days) across the Department of Medical Oncology and Hematology. Observed and predicted proportions, as well as 95% prediction intervals are shown for the most recent 17 months. Multivariable linear models (bottom table) were fitted to the monthly proportions or counts between January 2019 and May 2020, adjusting for the performance in the previous month (lagged proportion/count).

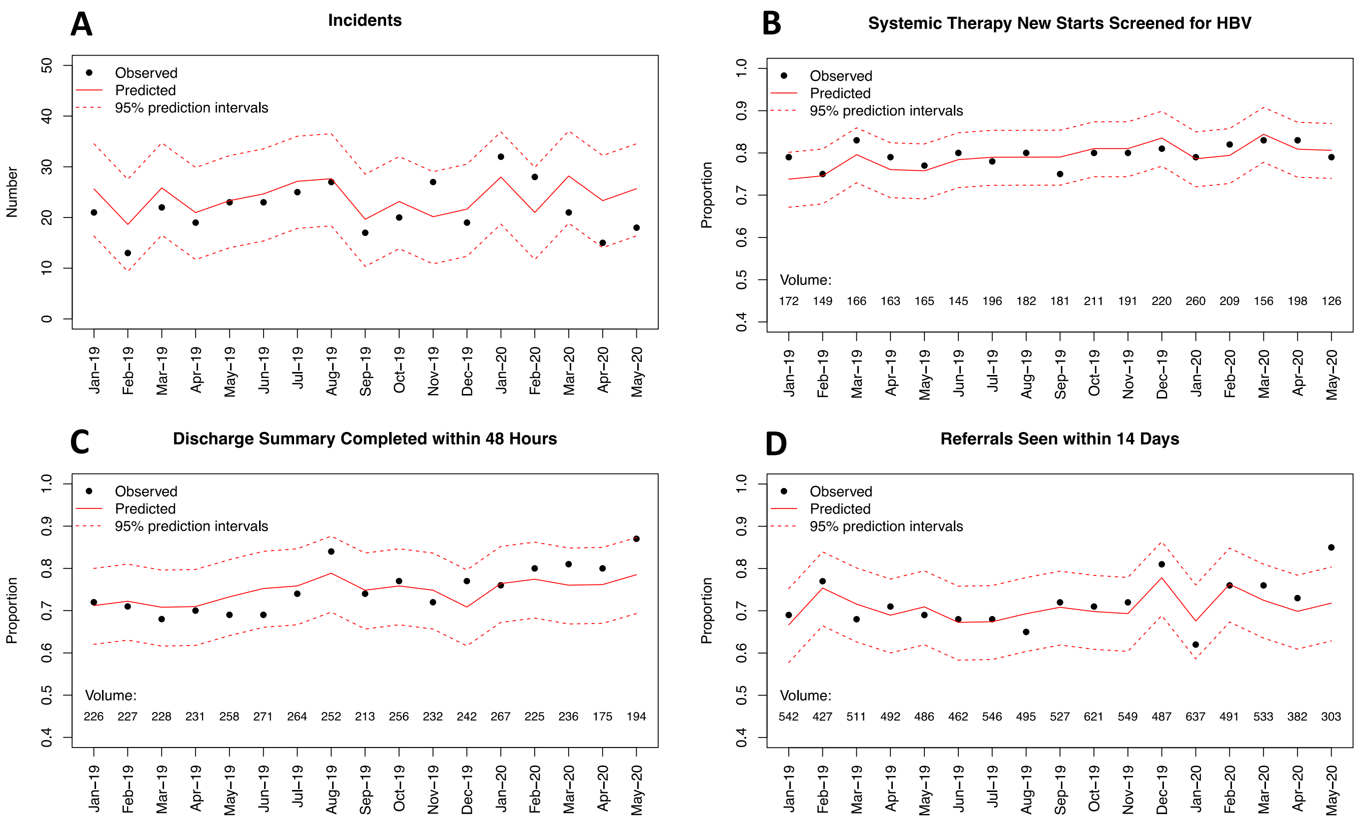


| Covariate | Incidents | | HBV Screens | | Discharge Summary | | Referrals | |
|---|---|---|---|---|---|---|---|---|
| | Estimate (95%) | p-value | Estimate (95%) | p-value | Estimate (95%) | p-value | Estimate (95%) | p-value |
| **Period** | | 0.064 | | 0.66 | | 0.15 | | 0.064 |
| Pre-Covid-19 | reference | | reference | | reference | | reference | |
| Covid-19 | -7.6 (-15.65,0.45) | | -0.01 (-0.07,0.04) | | 0.06 (-0.02,0.15) | | 0.07 (-4.2e-03,0.15) | |
| **Lagged proportion/count** | 0.06 (-0.42,0.54) | 0.82 | 0.39 (-0.09,0.87) | 0.11 | -0.07 (-0.55,0.41) | 0.78 | -0.16 (-0.79,0.48) | 0.63 |

© 2020 Berlin A et al. *JAMA Oncology.*

**eFigure 6.** Physician satisfaction survey individual responses, stratified by staff or trainee (resident, fellow) role (n=88 and 40, respectively).

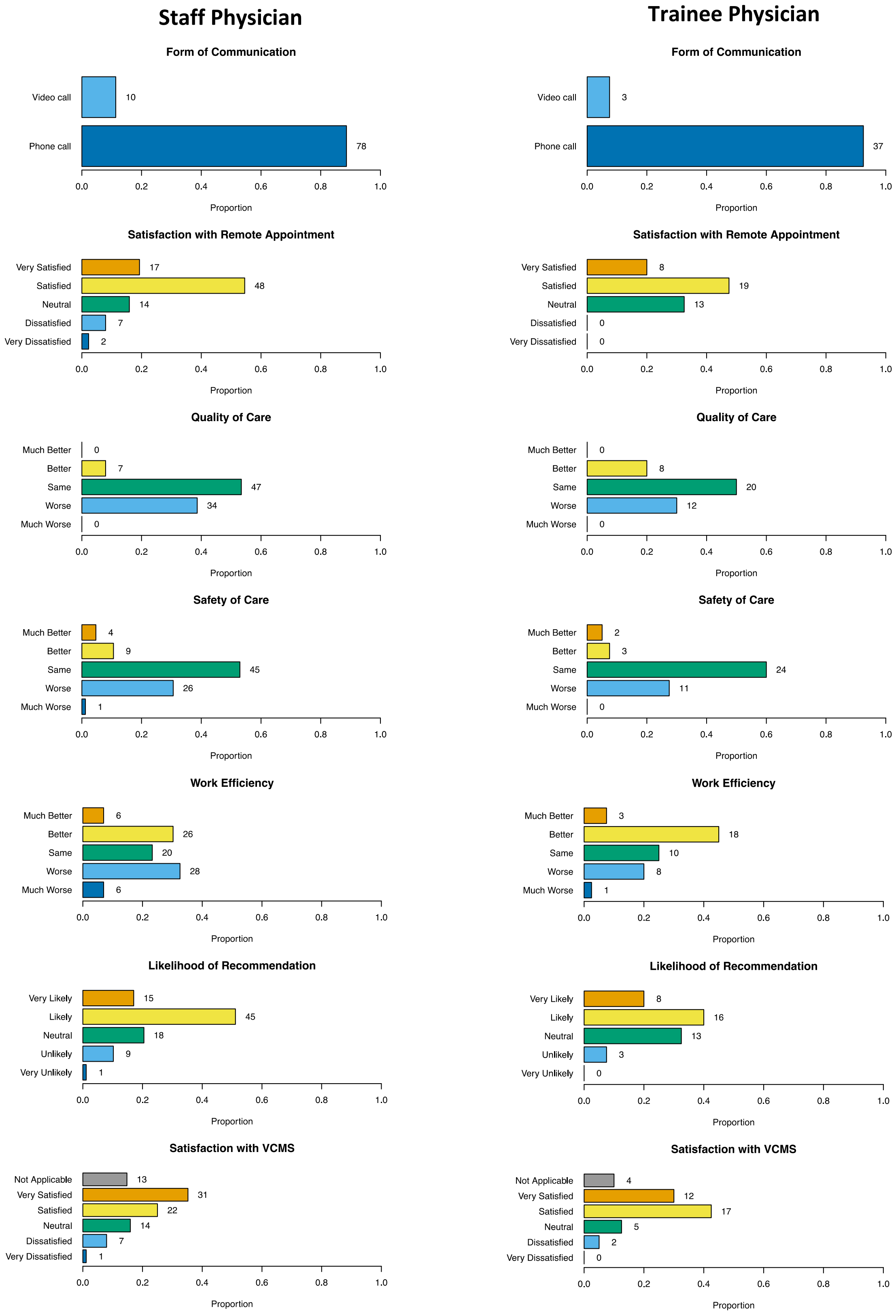


© 2020 Berlin A et al. *JAMA Oncology.*

**eFigure 7.** Physician satisfaction survey individual responses (n=128), stratified by VC communication type.

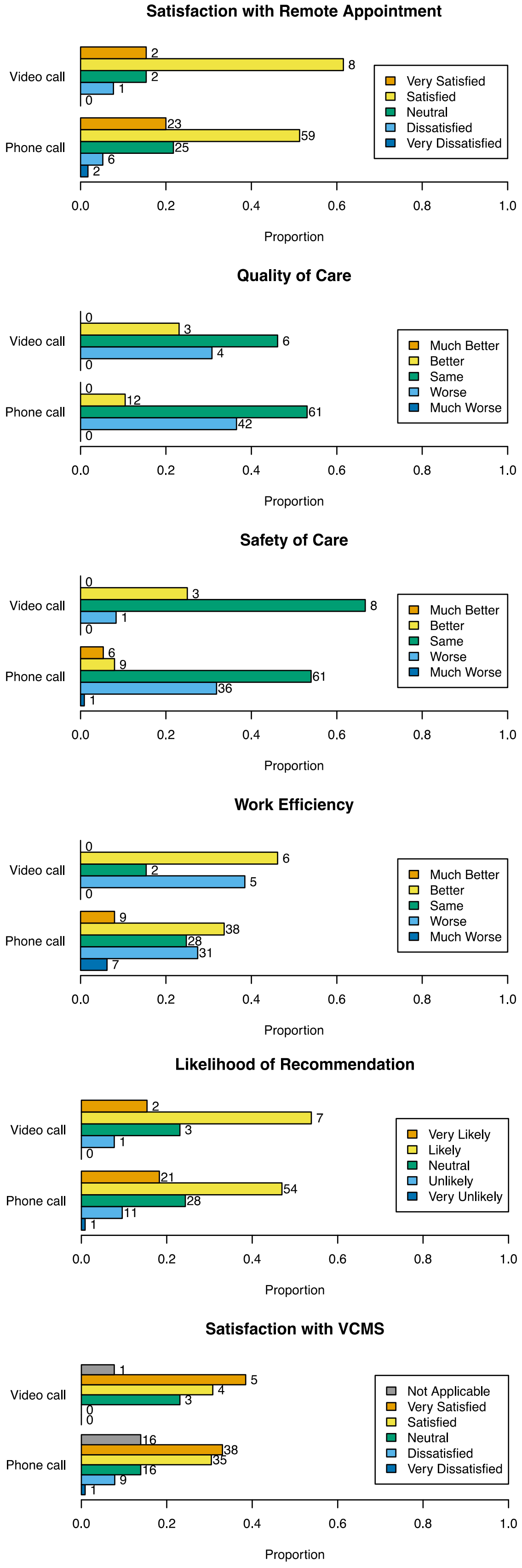


© 2020 Berlin A et al. *JAMA Oncology.*

## Supplementary Tables

**eTable 1.** Project direct costs.

| | | March 11 - April 3 | | April 4 - May 3 | | May 4 - May 22 | |
|---|---|---|---|---|---|---|---|
| | | Nominal Costs | Time allocation | Nominal Costs | Time allocation | Nominal Costs | Time allocation |
| **Human Resources** | Medical director (stipend) | 2,142.9 | 0.5 | 2,381.0 | 0.5 | 1,785.7 | 0.5 |
| | Design director | 5,535.7 | 0.5 | 6,150.8 | 0.5 | 4,613.1 | 0.5 |
| | Project lead | 7,971.4 | 1 | 8,857.1 | 1 | 6,642.9 | 1 |
| | Designer 1 | 3,100.0 | 0.5 | 3,444.4 | 0.5 | 2,583.3 | 0.5 |
| | Designer 2 | 4,207.1 | 0.5 | 4,674.6 | 0.5 | 3,506.0 | 0.5 |
| | Intern student | 3,600.0 | 1.0 | 4,000.0 | 1.0 | 3,000.0 | 1.0 |
| | IT project director | 11,071.4 | 1 | 9,841.3 | 0.8 | 7,381.0 | 0.8 |
| | Software architect | 9,357.1 | 1 | 10,396.8 | 1.0 | 4,678.6 | 0.6 |
| | Senior developer 1 | 6,571.4 | 0.8 | 7,301.6 | 0.8 | 3,422.6 | 0.5 |
| | Senior developer 2 | 4,107.1 | 0.5 | 4,563.5 | 0.5 | 2,053.6 | 0.3 |
| | Lead analyst | 6,857.1 | 1 | 3,809.5 | 0.5 | 2,857.1 | 0.5 |
| | QA Analyst 1 | 4,642.9 | 1 | 5,158.7 | 1.0 | 1,934.5 | 0.5 |
| | QA Analyst 2 | 4,642.9 | 1 | 2,579.4 | 0.5 | 773.8 | 0.2 |
| | QA Analyst 3 | 2,321.4 | 0.5 | 1,031.7 | 0.2 | - | - |
| | ***HR subtotal*** | ***76,128.6*** | | ***74,190.5*** | | ***45,232.1*** | |
| **Software/licences** | SoGoSurvey | 1,342.6 | | 1,342.6 | | 1,342.6 | |
| | Lucidchart | 11.0 | | 11.0 | | 11.0 | |
| | Whimsical | 10.0 | | 10.0 | | 10.0 | |
| | Notion | 8.0 | | 8.0 | | 8.0 | |
| | Figma | 12.0 | | 12.0 | | 12.0 | |
| | Visio | 50.0 | | 50.0 | | 50.0 | |
| | Appache Web Server (&) | - | | - | | - | |
| | PHP Scripting (&) | - | | - | | - | |
| | Jquery (&) | - | | - | | - | |
| | Enterprise Oracle Database (#) | 200.0 | | 200.0 | | 200.0 | |
| | Enterprise PowerBI (#) | 500.0 | | 500.0 | | 500.0 | |
| | ***Software subtotal*** | ***2,133.6*** | | ***2,133.6*** | | ***2,133.6*** | |
| **Hardware** | Virtual Server | 110.0 | | 110.0 | | 110.0 | |
| | Storage (1TB) | 85.0 | | 85.0 | | 85.0 | |
| | ***Hardware subtotal*** | ***195.0*** | | ***195.0*** | | ***195.0*** | |
| | ***Subtotal*** | ***78,457.2*** | | ***76,519.1*** | | ***47,560.7*** | |
| | **TOTAL project cost** | **202,537.0** | | | | | |

& Open source
# Shared with other applications and institutional initiatives
Note: time allocation during the corresponding periods reflects the proportion of working hours dedicated to this initiative in relation to a standard contract (e.g. 37.5 hours/week), without payment of overtime hours.

© 2020 Berlin A et al. *JAMA Oncology.*

**eTable 2.** Multivariable log-linear model of ambulatory visits at PM over time. Three periods are defined: period 1 (pre-pandemic; February 18 to March 10, 2020), period 2 (March 11 to April 19, 2020), and period 3 (April 20 to May 22, 2020). Model is fitted to the number of visits (on log scale), and number of visits in the previous day (on log scale) to account for the correlation over time. Weekends and statutory holidays were excluded due to small numbers.

| Covariate | Estimate (95%CI) | p-value | Global p-value |
|---|---|---|---|
| Period | | | **<0.001** |
| Feb 18 - Mar 10 | reference | | |
| Mar 11 - Apr 19 | -0.14 (-0.22,-0.06) | **<0.001** | |
| Apr 20 - May 22 | -0.03 (-0.1,0.03) | 0.36 | |
| Weekday | | | **<0.001** |
| Monday | reference | | |
| Tuesday | 0.04 (-0.07,0.14) | 0.5 | |
| Wednesday | 0.01 (-0.11,0.14) | 0.84 | |
| Thursday | -0.05 (-0.17,0.07) | 0.44 | |
| Friday | -0.39 (-0.5,-0.28) | **<0.001** | |
| Lagged count | 0.29 (0.07,0.51) | | **0.0095** |

**eTable 3.** Multivariable log-linear model of ambulatory visits over time at two comparable tertiary-hospitals within 2 kilometers distance from PM. Three periods are defined: period 1 (pre-pandemic; February 18 to March 10, 2020), period 2 (March 11 to April 19, 2020), and period 3 (April 20 to May 22, 2020). Model is fitted to the number of visits (on log scale), and number of visits in the previous day (on log scale) to account for the correlation over time. Weekends and statutory holidays were excluded analyses due to small numbers.

| | Hospital A | | | Hospital B | | |
|---|---|---|---|---|---|---|
| Covariate | Estimate (95%CI) | p-value | Global p-value | Estimate (95%CI) | p-value | Global p-value |
| Period | | | **<0.001** | | | **0.0016** |
| Feb 18 - Mar 10 | reference | | | reference | | |
| Mar 11 - Apr 19 | -0.19 (-0.28,-0.1) | **<0.001** | | -0.21 (-0.34,-0.09) | **<0.001** | |
| Apr 20 - May 22 | -0.12 (-0.2,-0.04) | **0.0044** | | -0.22 (-0.36,-0.09) | **0.0014** | |
| Weekday | | | **<0.001** | | | **<0.001** |
| Monday | reference | | | reference | | |
| Tuesday | 0.11 (0.03,0.2) | **0.011** | | -0.23 (-0.36,-0.1) | **<0.001** | |
| Wednesday | 6.8e-03 (-0.1,0.12) | 0.9 | | -0.3 (-0.44,-0.16) | **<0.001** | |
| Thursday | -0.01 (-0.12,0.09) | 0.83 | | -0.27 (-0.4,-0.14) | **<0.001** | |
| Friday | -0.3 (-0.4,-0.2) | **<0.001** | | -0.69 (-0.82,-0.55) | **<0.001** | |
| Lagged count | 0.46 (0.26,0.66) | | **<0.001** | 0.55 (0.36,0.74) | | **<0.001** |

© 2020 Berlin A et al. *JAMA Oncology.*

**eTable 4.** Survey completion rates.

Patients

| Method | Sent | Delivered | Invitation read | Survey opened | Opted out | Complete responses | Incomplete responses |
|---|---|---|---|---|---|---|---|
| **SMS** | 9,493 | 9,488 | 21.35% | 21.35% | 0.57% | 1,806 | 152 |
| **e-mail** | 5,151 | 4,855 | 69.08% | 36.64% | 0.84% | 1,701 | 54 |
| **TOTAL** | 14,644 | 14,343 | 37.50% | 26.53% | 0.66% | 3,507 | 206 |

Physicians

| Method | Sent | Delivered | Invitation read | Survey opened | Opted out | Complete responses | Incomplete responses |
|---|---|---|---|---|---|---|---|
| **e-mail** | 536 | 525 | 78.86% | 32.76% | 1.33% | 161 | 4 |

Administrative assistants

| Method | Sent | Delivered | Invitation read | Survey opened | Opted out | Complete responses | Incomplete responses |
|---|---|---|---|---|---|---|---|
| **e-mail** | 263 | 252 | 67.46% | 33.73% | 4.37% | 70 | 4 |

Patient flow coordinators

| Method | Sent | Delivered | Invitation read | Survey opened | Opted out | Complete responses | Incomplete responses |
|---|---|---|---|---|---|---|---|
| **e-mail** | 149 | 142 | 69.08% | 40.85% | 1.41% | 53 | 3 |

© 2020 Berlin A et al. *JAMA Oncology.*

**eTable 5.** Summary of patient characteristics.

| Covariate | Responders[#] (n=2,738) | Non-responders (n=11,788) | Full Sample (n=14,526) |
|---|---|---|---|
| **Age (year)** | | | |
| Mean (sd) | 62.4 (12.9) | 61.0 (15.4) | 61.3 (15) |
| Median (min,max) | 64 (19,97) | 63 (4,100) | 63 (4,100) |
| **Gender** | | | |
| Female | 1418 (52) | 5793 (49) | 7212 (50) |
| Male | 1320 (48) | 5994 (51) | 7313 (50) |
| Unknown | 0 (0) | 1 (0) | 1 (0) |
| **Income (Canadian dollar)** | | | |
| Mean (sd) | 64816.5 (27475.4) | 61976.9 (28083.5) | 62513.7 (27990.7) |
| Median (min,max) | 60794 (11072,282826) | 57592 (11072,366798) | 58292 (11072,366798) |
| Missing | 13 | 100 | 113 |
| **Income Quintile** | | | |
| 1 | 407 (15) | 2108 (18) | 2515 (17) |
| 2 | 431 (16) | 2267 (19) | 2698 (19) |
| 3 | 499 (18) | 2127 (18) | 2626 (18) |
| 4 | 518 (19) | 2106 (18) | 2624 (18) |
| 5 | 870 (32) | 3080 (26) | 3950 (27) |
| Unknown | 13 (0) | 95 (1) | 108 (1) |
| Missing | 0 | 5 | 5 |
| **Driving Distance (km)** | | | |
| <10 | 748 (27) | 3232 (28) | 3980 (28) |
| 10-25 | 602 (22) | 3055 (26) | 3657 (25) |
| 25-50 | 716 (26) | 3120 (27) | 3836 (27) |
| >50 | 664 (24) | 2331 (20) | 2995 (21) |
| Missing | 8 | 50 | 58 |
| **Driving Distance (km)** | | | |
| Mean (sd) | 76.2 (299.2) | 69.5 (304) | 70.8 (303.1) |
| Median (min,max) | 25.5 (0.3,4222.9) | 22.1 (0.3,4439.7) | 22.1 (0.3,4439.7) |
| Missing | 8 | 50 | 58 |
| **Driving time (min)** | | | |
| Mean (sd) | 59.6 (177.7) | 55.6 (180) | 56.3 (179.6) |
| Median (min,max) | 31.1 (1.3,2548) | 29 (1.3,2663.4) | 29.6 (1.3,2663.4) |
| Missing | 8 | 50 | 58 |
| **Residential Instability Quintile** | | | |
| 1 | 450 (17) | 1798 (15) | 2248 (16) |
| 2 | 464 (17) | 1828 (16) | 2292 (16) |
| 3 | 417 (15) | 1774 (15) | 2191 (15) |
| 4 | 505 (19) | 2172 (19) | 2677 (19) |
| 5 | 874 (32) | 4068 (35) | 4942 (34) |
| Missing | 28 | 148 | 176 |
| **Residential Instability Scores** | | | |
| Mean (sd) | 0.3 (1.2) | 0.4 (1.2) | 0.4 (1.2) |
| Median (min,max) | -0.1 (-1.5,4.2) | 0 (-1.7,4.2) | 0 (-1.7,4.2) |
| Missing | 28 | 148 | 176 |
| | | | |

© 2020 Berlin A et al. *JAMA Oncology.*

| | | | |
|---|---|---|---|
| **Economic Dependency Quintiles** | | | |
| 1 | 712 (26) | 2993 (26) | 3705 (26) |
| 2 | 539 (20) | 2398 (21) | 2937 (20) |
| 3 | 495 (18) | 2127 (18) | 2622 (18) |
| 4 | 458 (17) | 2102 (18) | 2560 (18) |
| 5 | 506 (19) | 2020 (17) | 2526 (18) |
| Missing | 28 | 148 | 176 |
| **Economic Dependency Scores** | | | |
| Mean (sd) | -0.1 (1) | -0.1 (1) | -0.1 (1) |
| Median (min,max) | -0.2 (-2.9,6) | -0.2 (-2.9,8.9) | -0.2 (-2.9,8.9) |
| Missing | 28 | 148 | 176 |
| **Ethno Cultural Composition Quintiles** | | | |
| 1 | 160 (6) | 582 (5) | 742 (5) |
| 2 | 355 (13) | 1137 (10) | 1492 (10) |
| 3 | 550 (20) | 2097 (18) | 2647 (18) |
| 4 | 801 (30) | 3434 (30) | 4235 (30) |
| 5 | 844 (31) | 4390 (38) | 5234 (36) |
| Missing | 28 | 148 | 176 |
| **Ethno Cultural Composition Score** | | | |
| Mean (sd) | 0.4 (1) | 0.5 (1) | 0.5 (1) |
| Median (min,max) | 0.2 (-1.3,5.2) | 0.4 (-1.3,5.4) | 0.3 (-1.3,5.4) |
| Missing | 28 | 148 | 176 |
| **Situational Vulnerability Quintiles** | | | |
| 1 | 1009 (37) | 3932 (34) | 4941 (34) |
| 2 | 636 (23) | 2522 (22) | 3158 (22) |
| 3 | 437 (16) | 1990 (17) | 2427 (17) |
| 4 | 347 (13) | 1714 (15) | 2061 (14) |
| 5 | 281 (10) | 1482 (13) | 1763 (12) |
| Missing | 28 | 148 | 176 |
| **Situational Vulnerability Scores** | | | |
| Mean (sd) | -0.4 (0.7) | -0.3 (0.8) | -0.3 (0.7) |
| Median (min,max) | -0.5 (-2,6.4) | -0.4 (-2.1,9.7) | -0.5 (-2.1,9.7) |
| Missing | 28 | 148 | 176 |

[#] 2,738 responders correspond to an individual patient, while 453, 111, 30, 8 and 3 patients had respectively 2, 3, 4, 5 and 6 independent surveys and recorded responses during the study period (not included in the analyses).

© 2020 Berlin A et al. *JAMA Oncology.*

**eTable 6.** Patient satisfaction stratified by VC modality. Responses to corresponding survey questions (Q): Q2, ‘Overall, how satisfied were you with your remote appointment?’; Q3, ‘How did your remote appointment compare to your usual in-person visit?’; and Q4, ‘How likely would you be to request another remote appointment?’. Results shown based on the original 5-level Likert scale (top) and for collapsed 3-level Likert scale (bottom). Absolute numbers and percentages (in parenthesis) are shown in each corresponding cell.

## 5-level Likert scale

| | Full Sample (n=2738)[#] | Phone call (n=2251) | Video call (n=431) | p-value |
|---|---|---|---|---|
| Q2 Satisfaction | | | | 0.0087 |
| Very Dissatisfied | 259 (10) | 235 (10) | 24 (6) | |
| Dissatisfied | 63 (2) | 47 (2) | 16 (4) | |
| Neutral | 155 (6) | 131 (6) | 24 (6) | |
| Satisfied | 788 (29) | 657 (29) | 131 (30) | |
| Very Satisfied | 1411 (53) | 1175 (52) | 236 (55) | |
| Missing | 6 | 6 | 0 | |
| Q3 Comparison | | | | 0.0062 |
| Much Worse | 41 (2) | 33 (1) | 8 (2) | |
| Worse | 354 (13) | 295 (13) | 59 (14) | |
| Same | 1771 (67) | 1514 (68) | 257 (60) | |
| Better | 311 (12) | 242 (11) | 69 (16) | |
| Much Better | 179 (7) | 143 (6) | 36 (8) | |
| Missing | 26 | 24 | 2 | |
| Q4 Likelihood | | | | <0.001 |
| Very Unlikely | 97 (4) | 84 (4) | 13 (3) | |
| Unlikely | 141 (5) | 126 (6) | 15 (3) | |
| Neutral | 622 (23) | 551 (25) | 71 (17) | |
| Likely | 938 (35) | 787 (35) | 151 (35) | |
| Very Likely | 870 (33) | 691 (31) | 179 (42) | |
| Missing | 14 | 12 | 2 | |

## 3-level Likert scale

| | Full Sample (n=2738)[#] | Phone call (n=2251) | Video call (n=431) | p-value |
|---|---|---|---|---|
| Satisfaction | | | | 0.15 |
| Dissatisfied | 322 (12) | 282 (13) | 40 (9) | |
| Neutral | 155 (6) | 131 (6) | 24 (6) | |
| Satisfied | 2199 (82) | 1832 (82) | 367 (85) | |
| Missing | 6 | 6 | 0 | |
| Comparison | | | | 0.001 |
| Worse | 395 (15) | 328 (15) | 67 (16) | |
| Same | 1771 (67) | 1514 (68) | 257 (60) | |
| Better | 490 (18) | 385 (17) | 105 (24) | |
| Missing | 26 | 24 | 2 | |
| Likelihood | | | | <0.001 |
| Unlikely | 238 (9) | 210 (9) | 28 (7) | |
| Neutral | 622 (23) | 551 (25) | 71 (17) | |
| Likely | 1808 (68) | 1478 (66) | 330 (77) | |
| Missing | 14 | 12 | 2 | |

# - 56 patients from the full sample had missing communication type (Q1).

© 2020 Berlin A et al. *JAMA Oncology.*

**eTable 7.** Complete survey responses: patients.

| | n=2738 |
|---|---|
| **Q1 Form of Communication** | |
| Phone call | 2251 (84) |
| Video call | 431 (16) |
| Missing | 56 |
| **Q2 Satisfaction with VC** | |
| Very Dissatisfied | 263 (10) |
| Dissatisfied | 64 (2) |
| Neutral | 156 (6) |
| Satisfied | 792 (29) |
| Very Satisfied | 1415 (53) |
| Missing | 48 |
| **Q3 Comparison** | |
| Much Worse | 46 (2) |
| Worse | 355 (13) |
| Same | 1773 (66) |
| Better | 314 (12) |
| Much Better | 181 (7) |
| Missing | 69 |
| **Q4 Likelihood of requesting** | |
| Very Unlikely | 101 (4) |
| Unlikely | 141 (5) |
| Neutral | 624 (23) |
| Likely | 943 (35) |
| Very Likely | 871 (32) |
| Missing | 58 |

**Q5 Likelihood of requesting another VC appointment and reasons**

| | Full Sample (n=2738) | Unlikely (n=242) | Neutral (n=624) | Likely (n=1814) |
|---|---|---|---|---|
| **Form of communication** | 803 (30) | 71 (29) | 154 (25) | 578 (32) |
| **Performance of technology** | 335 (12) | 29 (12) | 62 (10) | 244 (13) |
| **Quality of care** | 755 (28) | 90 (37) | 168 (27) | 497 (27) |
| **Convenience** | 1704 (64) | 45 (19) | 190 (30) | 1469 (81) |
| **Punctuality** | 637 (24) | 28 (12) | 59 (9) | 550 (30) |

© 2020 Berlin A et al. *JAMA Oncology.*

**eTable 8.** Multivariable analyses assessing the association of VC modality and socio-demographic factors with the patient-reported satisfaction with VC. The percentage with missing data and omitted from the analysis for the three outcomes 'Overall satisfaction with VC outcome', 'VC comparison to in-person', and 'Likelihood of requesting VC' were 3.47%, 4.19%, 3.70%, respectively.

| | ***Overall satisfaction with VC*** | | ***VC comparison to in-person*** | | ***Likelihood of requesting VC*** | |
|---|---|---|---|---|---|---|
| | **OR (95% CI)** | **p-value** | **OR (95% CI)** | **p-value** | **OR (95% CI)** | **p-value** |
| **Video call (Ref = Phone)** | 1.10 (0.92, 1.32) | 0.309 | 1.35 (1.09, 1.67) | **0.005** | 1.66 (1.39, 1.98) | **<0.001** |
| **Age (years)** | 1 (0.99, 1) | 0.281 | 1 (0.99, 1.01) | 0.921 | 1 (0.99, 1) | 0.334 |
| **Gender (Ref = Male)** | 1.20 (1.04, 1.38) | **0.014** | 1.02 (0.87, 1.20) | 0.774 | 0.94 (0.81, 1.08) | 0.352 |
| **Time of survey (week)** | 0.98 (0.95, 1.01) | 0.116 | 1.02 (0.98, 1.05) | 0.349 | 0.98 (0.95, 1.01) | 0.193 |
| **Income (per 10,000 dollars)** | 1.05 (1.01, 1.09) | **0.023** | 1.01 (0.97, 1.05) | 0.749 | 0.98 (0.95, 1.02) | 0.406 |
| **Residential instability** | 1.05 (0.98, 1.13) | 0.164 | 1.01 (0.94, 1.09) | 0.706 | 0.97 (0.9, 1.03) | 0.312 |
| **Economic dependency** | 0.99 (0.92, 1.06) | 0.784 | 1 (0.93, 1.07) | 0.933 | 1.01 (0.94, 1.08) | 0.786 |
| **Ethno-cultural composition** | 0.96 (0.9, 1.04) | 0.330 | 1.18 (1.09, 1.29) | **<0.001** | 0.89 (0.83, 0.96) | **0.002** |
| **Situational vulnerability** | 1.09 (0.97, 1.22) | 0.159 | 1.04 (0.91, 1.17) | 0.591 | 1.05 (0.93, 1.18) | 0.418 |

*Abbreviations: VC: virtual care; OR: odds ratio; CI: confidence interval.*
*Brand test p<0.05 for Overall satisfaction with VC on Age and Income, and Likelihood of requesting VC on Ethno-cultural composition and Situational vulnerability.*

© 2020 Berlin A et al. *JAMA Oncology.*

**eTable 9.** Complete survey responses: physicians.

| | n=128 |
|---|---|
| **Q1 Form of Communication** | |
| Phone call | 115 (90) |
| Video call | 13 (10) |
| **Q2 Satisfaction with VC** | |
| Very Dissatisfied | 2 (2) |
| Dissatisfied | 7 (5) |
| Neutral | 27 (21) |
| Satisfied | 67 (52) |
| Very Satisfied | 25 (20) |
| **Q3 Comparison - Quality of Care** | |
| Much Worse | 0 (0) |
| Worse | 46 (36) |
| Same | 67 (52) |
| Better | 15 (12) |
| Much Better | 0 (0) |
| **Q3 Comparison - Safety of Care** | |
| Much Worse | 1 (1) |
| Worse | 37 (30) |
| Same | 69 (55) |
| Better | 12 (10) |
| Much Better | 6 (5) |
| Missing | 3 |
| **Q3 Comparison - Work Efficiency** | |
| Much Worse | 7 (6) |
| Worse | 36 (29) |
| Same | 30 (24) |
| Better | 44 (35) |
| Much Better | 9 (7) |
| Missing | 2 |
| **Q4 Likelihood of recommending** | |
| Very Unlikely | 1 (1) |
| Unlikely | 12 (9) |
| Neutral | 31 (24) |
| Likely | 61 (48) |
| Very Likely | 23 (18) |

**Q5 Likelihood of recommending another VC appointment and reasons**

| | **Full Sample (n=128)** | **Unlikely (n=13)** | **Neutral (n=31)** | **Likely (n=84)** |
|---|---|---|---|---|
| **Form of communication** | 42 (33) | 5 (38) | 7 (23) | 30 (36) |
| **Performance of technology** | 33 (26) | 4 (31) | 2 (6) | 27 (32) |
| **Quality of care** | 43 (34) | 10 (77) | 17 (55) | 16 (19) |

| | | | | |
|---|---|---|---|---|
| **Safety of care** | 51 (40) | 7 (54) | 17 (55) | 27 (32) |
| **Efficiency** | 66 (52) | 7 (54) | 10 (32) | 49 (58) |
| **Convenience** | 73 (57) | 2 (15) | 3 (10) | 68 (81) |

| **Q6 Satisfaction with VCMS** | |
|---|---|
| Very Dissatisfied | 1 (1) |
| Dissatisfied | 9 (7) |
| Neutral | 19 (15) |
| Satisfied | 39 (30) |
| Very Satisfied | 43 (34) |
| Not Applicable | 17 (13) |

© 2020 Berlin A et al. *JAMA Oncology.*

**eTable 10.** Complete survey responses: administrative assistants.

| | n=52 |
|---|---|
| **Q1 Satisfaction with VCMS** | |
| Very Dissatisfied | 0 (0) |
| Dissatisfied | 1 (2) |
| Neutral | 20 (38) |
| Satisfied | 16 (31) |
| Very Satisfied | 10 (19) |
| Not Applicable | 5 (10) |
| **Q2 Work Efficiency** | |
| Much Worse | 1 (2) |
| Worse | 6 (12) |
| Same | 16 (31) |
| Better | 13 (25) |
| Much Better | 12 (23) |
| Not Applicable | 4 (8) |
| **Q2 Booking Errors** | |
| Much Worse | 1 (2) |
| Worse | 6 (12) |
| Same | 16 (32) |
| Better | 13 (26) |
| Much Better | 10 (20) |
| Not Applicable | 4 (8) |
| Missing | 2 |
| **Q2 Communication** | |
| Much Worse | 1 (2) |
| Worse | 6 (12) |
| Same | 17 (34) |
| Better | 14 (28) |
| Much Better | 8 (16) |
| Not Applicable | 4 (8) |
| Missing | 2 |
| **Q3 Comparison** | |
| Much Worse | 0 (0) |
| Worse | 4 (8) |
| Same | 13 (25) |
| Better | 9 (17) |
| Much Better | 15 (29) |
| Not working remotely | 11 (21) |
| **Q4 Likelihood** | |
| Very Unlikely | 0 (0) |
| Unlikely | 6 (12) |
| Neutral | 10 (19) |
| Likely | 13 (25) |
| Very Likely | 19 (37) |
| Not Applicable | 4 (8) |

© 2020 Berlin A et al. *JAMA Oncology.*

**Q5 Likelihood of recommending VCMS and reasons**

| | Full Sample (n=52) | Unlikely (n=6) | Neutral (n=10) | Likely (n=32) |
|---|---|---|---|---|
| **Performance of technology** | 16 (31) | 3 (50) | 2 (20) | 11 (34) |
| **Efficiency** | 27 (52) | 4 (67) | 5 (50) | 18 (56) |
| **Communication with physician** | 26 (50) | 4 (67) | 3 (30) | 19 (59) |
| **Convenience** | 29 (56) | 3 (50) | 3 (30) | 23 (72) |

© 2020 Berlin A et al. *JAMA Oncology.*

**eTable 11.** Complete survey responses: patient flow coordinators.

| | n=35 |
|---|---|
| **Q1 Satisfaction with VCMS** | |
| Very Dissatisfied | 1 (3) |
| Dissatisfied | 1 (3) |
| Neutral | 2 (6) |
| Satisfied | 12 (34) |
| Very Satisfied | 12 (34) |
| Not Applicable | 7 (20) |
| **Q2 Work Efficiency** | |
| Much Worse | 0 (0) |
| Worse | 2 (6) |
| Same | 8 (24) |
| Better | 7 (21) |
| Much Better | 10 (29) |
| Not Applicable | 7 (21) |
| Missing | 1 |
| **Q2 Clarity and Completeness** | |
| Much Worse | 1 (3) |
| Worse | 5 (15) |
| Same | 5 (15) |
| Better | 5 (15) |
| Much Better | 11 (32) |
| Not Applicable | 7 (21) |
| Missing | 1 |
| **Q2 Communication** | |
| Much Worse | 0 (0) |
| Worse | 4 (12) |
| Same | 8 (24) |
| Better | 7 (21) |
| Much Better | 6 (18) |
| Not Applicable | 8 (24) |
| Missing | 2 |
| **Q3 Comparison** | |
| Much Worse | 0 (0) |
| Worse | 1 (3) |
| Same | 2 (6) |
| Better | 4 (11) |
| Much Better | 7 (20) |
| Not working remotely | 21 (60) |
| **Q4 Likelihood** | |
| Very Unlikely | 0 (0) |
| Unlikely | 2 (6) |
| Neutral | 1 (3) |
| Likely | 8 (23) |
| Very Likely | 17 (49) |
| Not Applicable | 7 (20) |

© 2020 Berlin A et al. *JAMA Oncology.*

**Q5 Likelihood of recommending VCMS and reasons**

| | Full Sample (n=35) | Unlikely (n=2) | Neutral (n=1) | Likely (n=25) |
|---|---|---|---|---|
| **Performance of technology** | 10 (29) | 0 (0) | 0 (0) | 10 (40) |
| **Efficiency** | 20 (57) | 1 (50) | 0 (0) | 19 (76) |
| **Communication with care team** | 9 (26) | 1 (50) | 1 (100) | 7 (28) |
| **Digital orders** | 15 (43) | 0 (0) | 0 (0) | 15 (60) |
| **Convenience** | 18 (51) | 0 (0) | 0 (0) | 18 (72) |

© 2020 Berlin A et al. *JAMA Oncology.*

**eTable 12.** Univariable ordinal logistic regressions to assess differences between responses in the two survey rounds (5 weeks apart) among providers.

**Physicians**

| Model | | OR (95% CI) | p-value |
|---|---|---|---|
| Overall satisfaction | Round 2 (Ref = 1) | 0.86 (0.52, 1.45) | 0.58 |
| Quality of care | Round 2 (Ref = 1) | 0.88 (0.51, 1.55) | 0.67 |
| Safety of care | Round 2 (Ref = 1) | 0.52 (0.29, 0.93) | **0.03** |
| Work efficiency | Round 2 (Ref = 1) | 0.69 (0.41, 1.14) | 0.15 |
| Likelihood (VC) | Round 2 (Ref = 1) | 0.98 (0.59, 1.66) | 0.95 |
| Satisfaction (VCMS) | Round 2 (Ref = 1) | 1.56 (0.9, 2.7) | 0.12 |

**Administrative assistants**

| Model | | OR (95% CI) | p-value |
|---|---|---|---|
| Work efficiency | Round 2 (Ref = 1) | 0.74 (0.31, 1.8) | 0.51 |
| Booking errors | Round 2 (Ref = 1) | 0.86 (0.33, 2.24) | 0.76 |
| Communication | Round 2 (Ref = 1) | 0.91 (0.4, 2.11) | 0.83 |
| Comparison | Round 2 (Ref = 1) | 0.68 (0.28, 1.65) | 0.39 |
| Likelihood (VCMS) | Round 2 (Ref = 1) | 0.76 (0.33, 1.73) | 0.51 |

**Patient flow coordinators**

| Model | | OR | p-value |
|---|---|---|---|
| Work efficiency | Round 2 (Ref = 1) | 1.14 (0.44, 2.95) | 0.78 |
| Clarity and completeness | Round 2 (Ref = 1) | 1.12 (0.41, 3.1) | 0.82 |
| Communication | Round 2 (Ref = 1) | 1.54 (0.58, 4.13) | 0.39 |
| Comparison | Round 2 (Ref = 1) | 0.88 (0.34, 2.26) | 0.79 |
| Likelihood (VCMS) | Round 2 (Ref = 1) | 1.23 (0.42, 3.59) | 0.70 |

© 2020 Berlin A et al. *JAMA Oncology.*

**References**


1. Stickdorn M, Hormess M, Lawrence A, Schneider J. This Is Service Design Doing: Applying Service Design Thinking in the Real World: A Practitioners' Handbook. Chapter 2. O'Reilly Media, Incorporated; 2018.
2. Eisermann R et al. "The Double Diamond". Design Council UK (2004) Retrieved Jun 24 2020: https://www.designcouncil.org.uk/news-opinion/what-framework-innovation-design-councils-evolved-double-diamond
3. Stickdorn M, Hormess M, Lawrence A, Schneider J. This Is Service Design Doing: Applying Service Design Thinking in the Real World: A Practitioners' Handbook. Chapter 5. O'Reilly Media, Incorporated; 2018.
4. Altman M, Huang TTK, Breland JY. Design Thinking in Health Care. *Prev Chronic Dis*. 2018;15:180128.
5. Institute of Medicine, Committee on Quality of Health Care in America. Crossing the Quality Chasm: A New Health System for the 21st Century. National Academies Press; 2001.
6. Statistics Canada. 2019. "The Canadian Index of Multiple Deprivation". Statistics Canada Catalogue no. 45-20-0001.


© 2020 Berlin A et al. *JAMA Oncology.*
